# Supplementary material for: Immunoprotective Analysis of the NFA49590 Protein from Nocardia farcinica IFM 10152 Demonstrates Its Potential as a Vaccine Candidate
Source: Pathogens. 2022 Dec 7;11(12):1488. doi: 10.3390/pathogens11121488 (PMC9782307; doi:10.3390/pathogens11121488)
Supplement: Supplementary file 1 [file pathogens-11-01488-s001.zip › pathogens-2003876-supplementary.pdf]

Table S1. Secreted proteins in *N. farcinica* IFM10152 supernatants identified by LC–MS/MS.

No.	Accession	Score	Mass	Matches	Sequences	Name
1.	tr|Q5YPT1|Q5YPT1_NOCFA	8485	23078	376 (268)	11 (8)	Uncharacterized protein OS=Nocardia farcinica (strain IFM 10152) OX=247156 GN=NFA_49580 PE=4 SV=1
2.	tr|Q5YZF5|Q5YZF5_NOCFA	7802	23360	345 (252)	8 (7)	Uncharacterized protein OS=Nocardia farcinica (strain IFM 10152) OX=247156 GN=NFA_15900 PE=4 SV=1
3.	tr|Q5YMV0|Q5YMV0_NOCFA	6316	35256	320 (204)	20 (18)	Uncharacterized protein OS=Nocardia farcinica (strain IFM 10152) OX=247156 GN=NFA_56390 PE=4 SV=1
4.	tr|Q5YQC6|Q5YQC6_NOCFA	4636	23555	123 (96)	8 (5)	Uncharacterized protein OS=Nocardia farcinica (strain IFM 10152) OX=247156 GN=NFA_47630 PE=4 SV=1
5.	tr|Q5Z3G5|Q5Z3G5_NOCFA	3856	66212	208 (134)	36 (25)	Putative esterase OS=Nocardia farcinica (strain IFM 10152) OX=247156 GN=NFA_1840 PE=4 SV=1
**6.**	**tr|Q5YPT0|Q5YPT0_NOCFA**	**3686**	**24085**	**181 (129)**	**9 (5)**	**Uncharacterized protein OS=Nocardia farcinica (strain IFM 10152) OX=247156 GN=NFA_49590 PE=4 SV=1**
7.	tr|Q5YNH2|Q5YNH2_NOCFA	2837	45784	100 (74)	21 (15)	Putative protease OS=Nocardia farcinica (strain IFM 10152) OX=247156 GN=NFA_54170 PE=4 SV=1
8.	tr|Q5YND0|Q5YND0_NOCFA	2622	47326	131 (82)	26 (19)	Uncharacterized protein OS=Nocardia farcinica (strain IFM 10152) OX=247156 GN=NFA_54590 PE=4 SV=1
9.	tr|Q5YPS7|Q5YPS7_NOCFA	2438	41001	124 (75)	15 (11)	Putative protease OS=Nocardia farcinica (strain IFM 10152) OX=247156 GN=NFA_49620 PE=4 SV=1
10.	tr|Q5YPE3|Q5YPE3_NOCFA	2353	21660	100 (84)	11 (10)	Uncharacterized protein OS=Nocardia farcinica (strain IFM 10152) OX=247156 GN=NFA_50960 PE=4 SV=1
11.	tr|Q5YU10|Q5YU10_NOCFA	1936	101582	131 (73)	42 (26)	Aconitate hydratase OS=Nocardia farcinica (strain IFM 10152) OX=247156 GN=acn PE=3 SV=1
12.	tr|Q5Z1X5|Q5Z1X5_NOCFA	1904	36221	89 (58)	12 (9)	Putative esterase OS=Nocardia farcinica (strain IFM 10152)

No.	Accession	Score	Mass	Matches	Sequences	Name
						OX=247156 GN=NFA_7210 PE=4 SV=1
13.	tr|Q5YWQ1|Q5YWQ1_NOCFA	1887	31786	115 (72)	15 (7)	Uncharacterized protein OS=Nocardia farcinica (strain IFM 10152) OX=247156 GN=NFA_25430 PE=4 SV=1
14.	tr|Q5YRM1|Q5YRM1_NOCFA	1828	43077	84 (55)	14 (12)	Uncharacterized protein OS=Nocardia farcinica (strain IFM 10152) OX=247156 GN=NFA_43210 PE=4 SV=1
15.	tr|Q5Z1D4|Q5Z1D4_NOCFA	1809	28336	132 (75)	14 (12)	Uncharacterized protein OS=Nocardia farcinica (strain IFM 10152) OX=247156 GN=NFA_9120 PE=4 SV=1
16.	tr|Q5YYS3|Q5YYS3_NOCFA	1653	37023	96 (62)	19 (13)	Putative transporter subunit OS=Nocardia farcinica (strain IFM 10152) OX=247156 GN=NFA_18220 PE=4 SV=1
17.	tr|Q5YNC4|Q5YNC4_NOCFA	1633	40201	90 (55)	17 (13)	Putative glycosyl hydrolase OS=Nocardia farcinica (strain IFM 10152) OX=247156 GN=NFA_54650 PE=4 SV=1
18.	sp|Q5YNI0|DNAK_NOCFA	1459	65720	126 (58)	33 (12)	Chaperone protein DnaK OS=Nocardia farcinica (strain IFM 10152) OX=247156 GN=dnaK PE=3 SV=1
19.	tr|Q5YTQ4|Q5YTQ4_NOCFA	1445	35960	69 (41)	18 (15)	Glyceraldehyde-3-phosphate dehydrogenase OS=Nocardia farcinica (strain IFM 10152) OX=247156 GN=gap PE=3 SV=1
20.	tr|Q5Z345|Q5Z345_NOCFA	1440	62086	86 (45)	29 (15)	Putative oxidoreductase OS=Nocardia farcinica (strain IFM 10152) OX=247156 GN=NFA_3040 PE=4 SV=1
21.	tr|Q5YZF6|Q5YZF6_NOCFA	1403	23715	106 (64)	8 (4)	Uncharacterized protein OS=Nocardia farcinica (strain IFM 10152) OX=247156 GN=NFA_15890 PE=4 SV=1
22.	tr|Q5YQ44|Q5YQ44_NOCFA	1365	47833	74 (51)	18 (10)	Uncharacterized protein OS=Nocardia farcinica (strain IFM 10152) OX=247156 GN=NFA_48450 PE=4 SV=1
23.	tr|Q5YQF8|Q5YQF8_NOCFA	1193	53136	53 (35)	13 (9)	Putative protease OS=Nocardia farcinica (strain IFM 10152) OX=247156 GN=NFA_47310 PE=4 SV=1
24.	tr|Q5YQC4|Q5YQC4_NOCFA	1116	25412	64 (41)	11 (8)	Uncharacterized protein OS=Nocardia farcinica (strain IFM

No.	Accession	Score	Mass	Matches	Sequences	Name
						10152) OX=247156 GN=NFA_47650 PE=4 SV=1
25.	tr|Q5Z326|Q5Z326_NOCFA	1105	47386	59 (39)	18 (11)	Uncharacterized protein OS=Nocardia farcinica (strain IFM 10152) OX=247156 GN=NFA_3230 PE=4 SV=1
26.	sp|Q5YXD6|BLAC_NOCFA	1090	32718	55 (34)	15 (10)	Beta-lactamase FAR-1 OS=Nocardia farcinica (strain IFM 10152) OX=247156 GN=bla PE=1 SV=2
27.	tr|Q5Z0G9|Q5Z0G9_NOCFA	1070	60315	84 (38)	28 (12)	Urocanate hydratase OS=Nocardia farcinica (strain IFM 10152) OX=247156 GN=hutU PE=3 SV=1
28.	tr|Q5Z3H8|Q5Z3H8_NOCFA	1059	77295	73 (41)	27 (16)	Uncharacterized protein OS=Nocardia farcinica (strain IFM 10152) OX=247156 GN=NFA_1710 PE=4 SV=1
29.	tr|Q5YMV5|Q5YMV5_NOCFA	968	22668	63 (35)	5 (4)	Uncharacterized protein OS=Nocardia farcinica (strain IFM 10152) OX=247156 GN=NFA_56340 PE=4 SV=1
30.	tr|Q5Z0T6|Q5Z0T6_NOCFA	956	28315	64 (35)	13 (7)	Uncharacterized protein OS=Nocardia farcinica (strain IFM 10152) OX=247156 GN=NFA_11100 PE=4 SV=1
31.	tr|Q5YRR4|Q5YRR4_NOCFA	954	32039	39 (24)	17 (11)	Putative electron transfer flavoprotein alpha subunit OS=Nocardia farcinica (strain IFM 10152) OX=247156 GN=NFA_42780 PE=4 SV=1
32.	tr|Q5YWX5|Q5YWX5_NOCFA	954	37142	44 (31)	14 (12)	Uncharacterized protein OS=Nocardia farcinica (strain IFM 10152) OX=247156 GN=NFA_24690 PE=4 SV=1
33.	tr|Q5YYV3|Q5YYV3_NOCFA	950	72206	82 (34)	29 (15)	Putative acyl-CoA synthetase OS=Nocardia farcinica (strain IFM 10152) OX=247156 GN=NFA_17920 PE=4 SV=1
34.	tr|Q5YZI0|Q5YZI0_NOCFA	906	42210	54 (40)	13 (12)	Putative lipase OS=Nocardia farcinica (strain IFM 10152) OX=247156 GN=NFA_15650 PE=4 SV=1
35.	tr|Q5Z178|Q5Z178_NOCFA	903	34534	29 (23)	7 (7)	Uncharacterized protein OS=Nocardia farcinica (strain IFM 10152) OX=247156 GN=NFA_9680 PE=4 SV=1
36.	sp|Q5YPG4|EFTU_NOCFA	862	43742	60 (28)	19 (12)	Elongation factor Tu OS=Nocardia farcinica (strain IFM

No.	Accession	Score	Mass	Matches	Sequences	Name
						10152) OX=247156 GN=tuf PE=3 SV=2
37.	tr|Q5YP09|Q5YP09_NOCFA	825	46950	106 (49)	21 (16)	Putative isocitrate lyase OS=Nocardia farcinica (strain IFM 10152) OX=247156 GN=aceA PE=4 SV=1
38.	tr|Q5Z3H7|Q5Z3H7_NOCFA	816	52538	37 (25)	11 (8)	Uncharacterized protein OS=Nocardia farcinica (strain IFM 10152) OX=247156 GN=NFA_1720 PE=4 SV=1
39.	tr|Q5YNH3|Q5YNH3_NOCFA	806	46411	55 (27)	12 (9)	Putative protease OS=Nocardia farcinica (strain IFM 10152) OX=247156 GN=NFA_54160 PE=4 SV=1
40.	tr|Q5Z296|Q5Z296_NOCFA	805	38234	58 (26)	15 (9)	Phosphate-binding protein PstS OS=Nocardia farcinica (strain IFM 10152) OX=247156 GN=pstS PE=3 SV=1
41.	tr|Q5YNX4|Q5YNX4_NOCFA	766	49924	71 (27)	30 (13)	Dihydrolipoyl dehydrogenase OS=Nocardia farcinica (strain IFM 10152) OX=247156 GN=NFA_52650 PE=3 SV=1
42.	tr|Q5YR72|Q5YR72_NOCFA	749	55666	80 (27)	19 (11)	Uncharacterized protein OS=Nocardia farcinica (strain IFM 10152) OX=247156 GN=NFA_44680 PE=4 SV=1
43.	tr|Q5YN16|Q5YN16_NOCFA	748	18489	20 (16)	6 (5)	Single-stranded DNA-binding protein OS=Nocardia farcinica (strain IFM 10152) OX=247156 GN=ssb PE=3 SV=1
44.	tr|Q5Z0S0|Q5Z0S0_NOCFA	738	34145	41 (23)	17 (9)	Uncharacterized protein OS=Nocardia farcinica (strain IFM 10152) OX=247156 GN=NFA_11260 PE=4 SV=1
45.	tr|Q5YP07|Q5YP07_NOCFA	736	39351	56 (31)	8 (7)	Uncharacterized protein OS=Nocardia farcinica (strain IFM 10152) OX=247156 GN=NFA_52320 PE=4 SV=1
46.	tr|Q5Z3V6|Q5Z3V6_NOCFA	723	46494	47 (21)	14 (7)	Putative phage head OS=Nocardia farcinica (strain IFM 10152) OX=247156 GN=NFA_430 PE=4 SV=1
47.	sp|Q5YPP8|SUCC_NOCFA	704	41160	47 (22)	16 (9)	Succinate--CoA ligase [ADP-forming] subunit beta OS=Nocardia farcinica (strain IFM 10152) OX=247156 GN=sucC PE=3 SV=1
48.	tr|Q5Z3N1|Q5Z3N1_NOCFA	697	26499	42 (25)	13 (11)	Putative short chain dehydrogenase OS=Nocardia farcinica

No.	Accession	Score	Mass	Matches	Sequences	Name
						(strain IFM 10152) OX=247156 GN=NFA_1180 PE=3 SV=1
49.	tr|Q5YUY9|Q5YUY9_NOCFA	661	19671	26 (20)	3 (3)	Uncharacterized protein OS=Nocardia farcinica (strain IFM 10152) OX=247156 GN=NFA_31550 PE=4 SV=1
50.	sp|Q5YSV6|PNP_NOCFA	656	80997	49 (25)	24 (11)	Polyribonucleotide nucleotidyltransferase OS=Nocardia farcinica (strain IFM 10152) OX=247156 GN=pnp PE=3 SV=1
51.	sp|Q5YQ30|ENO_NOCFA	653	44966	51 (24)	14 (9)	Enolase OS=Nocardia farcinica (strain IFM 10152) OX=247156 GN=eno PE=3 SV=1
52.	tr|Q5YPA3|Q5YPA3_NOCFA	650	15679	38 (22)	12 (9)	Uncharacterized protein OS=Nocardia farcinica (strain IFM 10152) OX=247156 GN=NFA_51360 PE=4 SV=1
53.	tr|Q5Z152|Q5Z152_NOCFA	619	58870	44 (19)	22 (6)	Putative acyl-CoA carboxylase beta subunit OS=Nocardia farcinica (strain IFM 10152) OX=247156 GN=NFA_9940 PE=4 SV=1
54.	sp|Q5YNB0|PCKG_NOCFA	617	68123	46 (24)	22 (13)	Phosphoenolpyruvate carboxykinase [GTP] OS=Nocardia farcinica (strain IFM 10152) OX=247156 GN=pckG PE=3 SV=1
55.	tr|Q5YRC6|Q5YRC6_NOCFA	605	49389	48 (25)	15 (8)	Uncharacterized protein OS=Nocardia farcinica (strain IFM 10152) OX=247156 GN=NFA_44140 PE=4 SV=1
56.	sp|Q5YPG3|EFG_NOCFA	601	77199	78 (31)	34 (16)	Elongation factor G OS=Nocardia farcinica (strain IFM 10152) OX=247156 GN=fusA PE=3 SV=1
57.	tr|Q5YSR9|Q5YSR9_NOCFA	599	33323	44 (30)	13 (8)	Uncharacterized protein OS=Nocardia farcinica (strain IFM 10152) OX=247156 GN=NFA_39240 PE=4 SV=1
58.	sp|Q5YPE1|RPOC_NOCFA	594	147275	107 (27)	62 (21)	DNA-directed RNA polymerase subunit beta' OS=Nocardia farcinica (strain IFM 10152) OX=247156 GN=rpoC PE=3 SV=1

No.	Accession	Score	Mass	Matches	Sequences	Name
59.	tr|Q5YZD2|Q5YZD2_NOCFA	581	102947	77 (21)	30 (11)	Pyruvate dehydrogenase E1 component OS=Nocardia farcinica (strain IFM 10152) OX=247156 GN=aceE PE=4 SV=1
60.	tr|Q5YTJ7|Q5YTJ7_NOCFA	574	58744	47 (22)	21 (8)	Putative alkaline phosphatase OS=Nocardia farcinica (strain IFM 10152) OX=247156 GN=NFA_36460 PE=4 SV=1
61.	tr|Q5YYV6|Q5YYV6_NOCFA	571	32899	26 (15)	9 (6)	Uncharacterized protein OS=Nocardia farcinica (strain IFM 10152) OX=247156 GN=NFA_17890 PE=4 SV=1
62.	tr|Q5Z1Y0|Q5Z1Y0_NOCFA	565	25595	52 (26)	9 (7)	Putative peptidase OS=Nocardia farcinica (strain IFM 10152) OX=247156 GN=NFA_7160 PE=4 SV=1
63.	tr|Q5Z1N3|Q5Z1N3_NOCFA	563	61699	68 (30)	26 (12)	Uncharacterized protein OS=Nocardia farcinica (strain IFM 10152) OX=247156 GN=NFA_8130 PE=4 SV=1
64.	tr|Q5YY91|Q5YY91_NOCFA	563	31372	35 (18)	13 (8)	Putative channel protein OS=Nocardia farcinica (strain IFM 10152) OX=247156 GN=NFA_20040 PE=4 SV=1
65.	tr|Q5YXF1|Q5YXF1_NOCFA	544	45000	27 (17)	10 (8)	Cytochrome P450 monooxygenase OS=Nocardia farcinica (strain IFM 10152) OX=247156 GN=NFA_22930 PE=3 SV=1
66.	tr|Q5YRP3|Q5YRP3_NOCFA	533	40895	37 (20)	13 (7)	Putative hydrolase OS=Nocardia farcinica (strain IFM 10152) OX=247156 GN=NFA_42990 PE=4 SV=1
67.	tr|Q5YR45|Q5YR45_NOCFA	532	22445	29 (14)	8 (6)	Uncharacterized protein OS=Nocardia farcinica (strain IFM 10152) OX=247156 GN=NFA_44950 PE=4 SV=1
68.	tr|Q5YZ78|Q5YZ78_NOCFA	528	53944	49 (17)	17 (7)	Glutamine synthetase OS=Nocardia farcinica (strain IFM 10152) OX=247156 GN=glnA2 PE=3 SV=1
69.	tr|Q5YN21|Q5YN21_NOCFA	523	20479	49 (23)	10 (5)	Uncharacterized protein OS=Nocardia farcinica (strain IFM 10152) OX=247156 GN=NFA_55680 PE=4 SV=1
70.	tr|Q5YN81|Q5YN81_NOCFA	514	54768	34 (18)	19 (9)	Putative hydrolase OS=Nocardia farcinica (strain IFM 10152) OX=247156 GN=NFA_55080 PE=4 SV=1

No.	Accession	Score	Mass	Matches	Sequences	Name
71.	tr|Q5YYZ6|Q5YYZ6_NOCFA	511	25196	24 (17)	7 (6)	Uncharacterized protein OS=Nocardia farcinica (strain IFM 10152) OX=247156 GN=NFA_17490 PE=4 SV=1
72.	tr|Q5Z1C3|Q5Z1C3_NOCFA	502	45343	55 (24)	18 (9)	Isocitrate dehydrogenase [NADP] OS=Nocardia farcinica (strain IFM 10152) OX=247156 GN=NFA_9230 PE=3 SV=1
73.	tr|Q5YQJ5|Q5YQJ5_NOCFA	482	139327	61 (15)	35 (8)	Putative 2-oxoglutarate dehydrogenase OS=Nocardia farcinica (strain IFM 10152) OX=247156 GN=sucA PE=4 SV=1
74.	tr|Q5Z3M2|Q5Z3M2_NOCFA	460	20599	35 (9)	11 (4)	Ferritin OS=Nocardia farcinica (strain IFM 10152) OX=247156 GN=NFA_1270 PE=3 SV=1
75.	tr|Q5YQY4|Q5YQY4_NOCFA	455	34268	22 (14)	11 (6)	Putative esterase OS=Nocardia farcinica (strain IFM 10152) OX=247156 GN=NFA_45560 PE=4 SV=1
76.	tr|Q5YZ12|Q5YZ12_NOCFA	451	68071	40 (16)	19 (6)	Putative serine/threonine protein kinase OS=Nocardia farcinica (strain IFM 10152) OX=247156 GN=NFA_17330 PE=4 SV=1
77.	tr|Q5Z235|Q5Z235_NOCFA	438	41193	38 (11)	17 (4)	Putative citrate synthase OS=Nocardia farcinica (strain IFM 10152) OX=247156 GN=NFA_6610 PE=3 SV=1
78.	sp|Q5YTQ5|PGK_NOCFA	436	42753	33 (10)	16 (5)	Phosphoglycerate kinase OS=Nocardia farcinica (strain IFM 10152) OX=247156 GN=pgk PE=3 SV=1
79.	tr|Q5YQQ1|Q5YQQ1_NOCFA	433	51500	35 (14)	17 (8)	Putative transcriptional regulator OS=Nocardia farcinica (strain IFM 10152) OX=247156 GN=NFA_46390 PE=4 SV=1
80.	sp|Q5YRX2|LEU3_NOCFA	425	35226	24 (15)	12 (6)	3-isopropylmalate dehydrogenase OS=Nocardia farcinica (strain IFM 10152) OX=247156 GN=leuB PE=3 SV=1
81.	tr|Q5YVE9|Q5YVE9_NOCFA	424	54995	38 (14)	14 (6)	Putative aldehyde dehydrogenase OS=Nocardia farcinica (strain IFM 10152) OX=247156 GN=NFA_29950 PE=3 SV=1
82.	sp|Q5YPE0|RPOB2_NOCFA	407	128926	62 (24)	37 (11)	DNA-directed RNA polymerase subunit beta 2 OS=Nocardia farcinica (strain IFM 10152) OX=247156 GN=rpoB2 PE=3

No.	Accession	Score	Mass	Matches	Sequences	Name
						SV=1
83.	tr|Q5YQ23|Q5YQ23_NOCFA	394	20431	17 (9)	5 (3)	Uncharacterized protein OS=Nocardia farcinica (strain IFM 10152) OX=247156 GN=NFA_48660 PE=4 SV=1
84.	tr|Q5Z0M7|Q5Z0M7_NOCFA	390	20068	16 (8)	5 (2)	Uncharacterized protein OS=Nocardia farcinica (strain IFM 10152) OX=247156 GN=NFA_11690 PE=4 SV=1
85.	tr|Q5Z0B4|Q5Z0B4_NOCFA	390	43517	36 (15)	11 (5)	Uncharacterized protein OS=Nocardia farcinica (strain IFM 10152) OX=247156 GN=NFA_12820 PE=4 SV=1
86.	tr|Q5YPP9|Q5YPP9_NOCFA	385	31273	64 (19)	14 (6)	Succinate--CoA ligase [ADP-forming] subunit alpha OS=Nocardia farcinica (strain IFM 10152) OX=247156 GN=sucD PE=3 SV=1
87.	tr|Q5YTP7|Q5YTP7_NOCFA	383	46288	37 (16)	18 (7)	Putative O-acetylhomoserine sulfhydrylase OS=Nocardia farcinica (strain IFM 10152) OX=247156 GN=metC PE=3 SV=1
88.	sp|Q5YRW2|ILVC_NOCFA	380	36137	39 (20)	16 (10)	Ketol-acid reductoisomerase (NADP(+)) OS=Nocardia farcinica (strain IFM 10152) OX=247156 GN=ilvC PE=3 SV=1
89.	tr|Q5YQ56|Q5YQ56_NOCFA	377	16041	23 (15)	6 (5)	Uncharacterized protein OS=Nocardia farcinica (strain IFM 10152) OX=247156 GN=NFA_48330 PE=4 SV=1
90.	tr|Q5Z0D9|Q5Z0D9_NOCFA	368	330021	100 (25)	59 (15)	Putative fatty acid synthase OS=Nocardia farcinica (strain IFM 10152) OX=247156 GN=NFA_12570 PE=4 SV=1
91.	tr|Q5YTS0|Q5YTS0_NOCFA	364	77099	46 (19)	21 (7)	Putative transketolase OS=Nocardia farcinica (strain IFM 10152) OX=247156 GN=NFA_35730 PE=3 SV=1
92.	tr|Q5Z078|Q5Z078_NOCFA	358	94184	37 (12)	23 (7)	Putative aminopeptidase OS=Nocardia farcinica (strain IFM 10152) OX=247156 GN=NFA_13180 PE=4 SV=1
93.	tr|Q5YNE3|Q5YNE3_NOCFA	358	41741	32 (15)	12 (6)	Uncharacterized protein OS=Nocardia farcinica (strain IFM

No.	Accession	Score	Mass	Matches	Sequences	Name
						10152) OX=247156 GN=NFA_54460 PE=4 SV=1
94.	tr|Q5YZA4|Q5YZA4_NOCFA	357	22500	34 (21)	4 (4)	Uncharacterized protein OS=Nocardia farcinica (strain IFM 10152) OX=247156 GN=NFA_16410 PE=4 SV=1
95.	tr|Q5YRX1|Q5YRX1_NOCFA	355	55601	33 (13)	21 (8)	D-3-phosphoglycerate dehydrogenase OS=Nocardia farcinica (strain IFM 10152) OX=247156 GN=serA PE=3 SV=1
96.	tr|Q5YZ26|Q5YZ26_NOCFA	355	34392	25 (17)	10 (6)	Uncharacterized protein OS=Nocardia farcinica (strain IFM 10152) OX=247156 GN=NFA_17190 PE=4 SV=1
97.	tr|Q5YU62|Q5YU62_NOCFA	354	32898	22 (10)	9 (3)	Uncharacterized protein OS=Nocardia farcinica (strain IFM 10152) OX=247156 GN=NFA_34310 PE=4 SV=1
98.	sp|Q9AFA6|CH602_NOCFA	350	56373	37 (11)	21 (9)	60 kDa chaperonin 2 OS=Nocardia farcinica (strain IFM 10152) OX=247156 GN=groL2 PE=3 SV=2
99.	tr|Q5Z338|Q5Z338_NOCFA	349	56243	39 (14)	22 (8)	Putative cholesterol oxidase OS=Nocardia farcinica (strain IFM 10152) OX=247156 GN=NFA_3110 PE=4 SV=1
100.	tr|Q5Z310|Q5Z310_NOCFA	348	83552	47 (15)	24 (9)	Putative penicillin-binding protein OS=Nocardia farcinica (strain IFM 10152) OX=247156 GN=NFA_3390 PE=4 SV=1
101.	tr|Q5YT06|Q5YT06_NOCFA	333	29474	24 (11)	12 (6)	Putative glutamate transporter glutamate-binding protein OS=Nocardia farcinica (strain IFM 10152) OX=247156 GN=NFA_38370 PE=4 SV=1
102.	tr|Q5YQ54|Q5YQ54_NOCFA	331	43110	29 (17)	14 (11)	Putative acyl-CoA thiolase OS=Nocardia farcinica (strain IFM 10152) OX=247156 GN=fadA10 PE=3 SV=1
103.	tr|Q5Z0L3|Q5Z0L3_NOCFA	328	30998	18 (10)	10 (5)	Uncharacterized protein OS=Nocardia farcinica (strain IFM 10152) OX=247156 GN=NFA_11830 PE=4 SV=1
104.	tr|Q5YMT1|Q5YMT1_NOCFA	322	89626	36 (10)	20 (4)	Uncharacterized protein OS=Nocardia farcinica (strain IFM 10152) OX=247156 GN=NFA_56580 PE=4 SV=1
105.	sp|Q5YQS7|SAHH_NOCFA	318	54182	43 (17)	22 (7)	Adenosylhomocysteinase OS=Nocardia farcinica (strain IFM

No.	Accession	Score	Mass	Matches	Sequences	Name
						10152) OX=247156 GN=ahcY PE=3 SV=1
106.	tr|Q5YR58|Q5YR58_NOCFA	313	38681	21 (8)	9 (3)	Uncharacterized protein OS=Nocardia farcinica (strain IFM 10152) OX=247156 GN=NFA_44820 PE=4 SV=1
107.	sp|Q5YYH6|SYFB_NOCFA	310	89266	24 (9)	19 (7)	Phenylalanine--tRNA ligase beta subunit OS=Nocardia farcinica (strain IFM 10152) OX=247156 GN=pheT PE=3 SV=1
108.	tr|Q5YQI1|Q5YQI1_NOCFA	309	41121	24 (15)	13 (8)	Putative malate dehydrogenase OS=Nocardia farcinica (strain IFM 10152) OX=247156 GN=NFA_47090 PE=3 SV=1
109.	tr|Q5Z241|Q5Z241_NOCFA	304	40432	21 (10)	11 (4)	Phosphoserine aminotransferase OS=Nocardia farcinica (strain IFM 10152) OX=247156 GN=serC PE=3 SV=1
110.	tr|Q5YT54|Q5YT54_NOCFA	302	21614	14 (8)	9 (4)	Putative alkylhydroperoxide reductase OS=Nocardia farcinica (strain IFM 10152) OX=247156 GN=ahpC PE=4 SV=1
111.	tr|Q5YX80|Q5YX80_NOCFA	293	32309	23 (11)	7 (3)	Uncharacterized protein OS=Nocardia farcinica (strain IFM 10152) OX=247156 GN=NFA_23640 PE=4 SV=1
112.	tr|Q5YTG2|Q5YTG2_NOCFA	286	33348	19 (6)	6 (3)	Uncharacterized protein OS=Nocardia farcinica (strain IFM 10152) OX=247156 GN=NFA_36810 PE=4 SV=1
113.	tr|Q5YP16|Q5YP16_NOCFA	282	26124	17 (10)	7 (3)	Uncharacterized protein OS=Nocardia farcinica (strain IFM 10152) OX=247156 GN=NFA_52230 PE=4 SV=1
114.	tr|Q5Z2Z8|Q5Z2Z8_NOCFA	278	40418	17 (8)	9 (4)	Putative protease OS=Nocardia farcinica (strain IFM 10152) OX=247156 GN=NFA_3510 PE=4 SV=1
115.	tr|Q5Z224|Q5Z224_NOCFA	276	31089	19 (10)	9 (4)	Putative oxidoreductase OS=Nocardia farcinica (strain IFM 10152) OX=247156 GN=NFA_6720 PE=4 SV=1
116.	tr|Q5YND1|Q5YND1_NOCFA	275	46731	22 (11)	7 (5)	Putative lipase OS=Nocardia farcinica (strain IFM 10152) OX=247156 GN=NFA_54580 PE=4 SV=1
117.	tr|Q5YN12|Q5YN12_NOCFA	273	57518	27 (9)	9 (4)	Uncharacterized protein OS=Nocardia farcinica (strain IFM

No.	Accession	Score	Mass	Matches	Sequences	Name
						10152) OX=247156 GN=NFA_55770 PE=4 SV=1
118.	tr|Q5YPP7|Q5YPP7_NOCFA	272	52247	14 (5)	4 (1)	Putative peptidase OS=Nocardia farcinica (strain IFM 10152) OX=247156 GN=NFA_49920 PE=4 SV=1
119.	tr|Q5YXU0|Q5YXU0_NOCFA	267	71261	33 (12)	17 (9)	Putative dehydrogenase OS=Nocardia farcinica (strain IFM 10152) OX=247156 GN=NFA_21550 PE=4 SV=1
120.	tr|Q5YRS8|Q5YRS8_NOCFA	266	10028	5 (3)	2 (1)	Uncharacterized protein OS=Nocardia farcinica (strain IFM 10152) OX=247156 GN=NFA_42640 PE=4 SV=1
121.	tr|Q5YV07|Q5YV07_NOCFA	261	130689	36 (14)	27 (10)	Putative magnesium chelatase OS=Nocardia farcinica (strain IFM 10152) OX=247156 GN=NFA_31370 PE=4 SV=1
122.	tr|Q5YU65|Q5YU65_NOCFA	256	26877	22 (10)	9 (4)	Uncharacterized protein OS=Nocardia farcinica (strain IFM 10152) OX=247156 GN=NFA_34280 PE=4 SV=1
123.	tr|Q5Z1J7|Q5Z1J7_NOCFA	253	46840	32 (12)	12 (5)	Putative protease OS=Nocardia farcinica (strain IFM 10152) OX=247156 GN=NFA_8490 PE=3 SV=1
124.	tr|Q5YMU9|Q5YMU9_NOCFA	249	75803	42 (8)	19 (6)	Putative peptidase OS=Nocardia farcinica (strain IFM 10152) OX=247156 GN=NFA_56400 PE=4 SV=1
125.	tr|Q5YYZ4|Q5YYZ4_NOCFA	243	27257	38 (19)	12 (7)	Putative lipoprotein OS=Nocardia farcinica (strain IFM 10152) OX=247156 GN=NFA_17510 PE=4 SV=1
126.	sp|Q5YYP5|HIS4_NOCFA	236	25600	11 (6)	5 (3)	1-(5-phosphoribosyl)-5-[(5-phosphoribosylamino)methylidene amino] imidazole-4-carboxamide isomerase OS=Nocardia farcinica (strain IFM 10152) OX=247156 GN=hisA PE=3 SV=1
127.	tr|Q5Z3G7|Q5Z3G7_NOCFA	230	37776	28 (8)	8 (4)	Putative mycolyltransferase OS=Nocardia farcinica (strain IFM 10152) OX=247156 GN=fbpB PE=4 SV=1
128.	tr|Q5YN37|Q5YN37_NOCFA	228	39498	40 (13)	13 (5)	Putative 1L-myo-inositol-1-phosphate synthase OS=Nocardia farcinica (strain IFM 10152) OX=247156 GN=NFA_55520

No.	Accession	Score	Mass	Matches	Sequences	Name
						PE=4 SV=1
129.	tr|Q5Z0G8|Q5Z0G8_NOCFA	228	54785	44 (15)	20 (8)	Histidine ammonia-lyase OS=Nocardia farcinica (strain IFM 10152) OX=247156 GN=hutH PE=3 SV=1
130.	tr|Q5YQW6|Q5YQW6_NOCFA	227	42872	30 (12)	14 (6)	Uncharacterized protein OS=Nocardia farcinica (strain IFM 10152) OX=247156 GN=NFA_45740 PE=4 SV=1
131.	tr|Q5YZ48|Q5YZ48_NOCFA	225	39862	32 (5)	8 (3)	Branched-chain-amino-acid aminotransferase OS=Nocardia farcinica (strain IFM 10152) OX=247156 GN=NFA_16970 PE=3 SV=1
132.	tr|Q5YPF3|Q5YPF3_NOCFA	224	39395	14 (4)	10 (3)	Uncharacterized protein OS=Nocardia farcinica (strain IFM 10152) OX=247156 GN=NFA_50860 PE=4 SV=1
133.	tr|Q5Z3R9|Q5Z3R9_NOCFA	223	67538	35 (8)	21 (3)	Putative serine/threonine protein kinase OS=Nocardia farcinica (strain IFM 10152) OX=247156 GN=NFA_800 PE=4 SV=1
134.	tr|Q5YSZ1|Q5YSZ1_NOCFA	218	20265	14 (9)	5 (3)	Peptidyl-prolyl cis-trans isomerase OS=Nocardia farcinica (strain IFM 10152) OX=247156 GN=NFA_38520 PE=3 SV=1
135.	tr|Q5YS41|Q5YS41_NOCFA	218	59076	23 (6)	16 (5)	Putative acyl-CoA synthetase OS=Nocardia farcinica (strain IFM 10152) OX=247156 GN=NFA_41510 PE=4 SV=1
136.	tr|Q5YTR9|Q5YTR9_NOCFA	215	40610	28 (12)	15 (7)	Transaldolase OS=Nocardia farcinica (strain IFM 10152) OX=247156 GN=tal PE=3 SV=1
137.	tr|Q5YVT7|Q5YVT7_NOCFA	214	57417	23 (10)	15 (7)	Phosphoenolpyruvate-protein phosphotransferase OS=Nocardia farcinica (strain IFM 10152) OX=247156 GN=ptsA PE=3 SV=1
138.	sp|Q5YP50|GPMA_NOCFA	212	27416	21 (7)	13 (4)	2,3-bisphosphoglycerate-dependent phosphoglycerate mutase OS=Nocardia farcinica (strain IFM 10152) OX=247156 GN=gpmA PE=3 SV=1

No.	Accession	Score	Mass	Matches	Sequences	Name
139.	sp|Q5YP70|HEM3_NOCFA	210	35599	17 (8)	10 (4)	Porphobilinogen deaminase OS=Nocardia farcinica (strain IFM 10152) OX=247156 GN=hemC PE=3 SV=1
140.	tr|Q5Z116|Q5Z116_NOCFA	207	33767	18 (7)	11 (3)	Putative monooxygenase OS=Nocardia farcinica (strain IFM 10152) OX=247156 GN=NFA_10300 PE=4 SV=1
141.	tr|Q5Z1B0|Q5Z1B0_NOCFA	201	37666	20 (8)	11 (4)	Tryptophan--tRNA ligase OS=Nocardia farcinica (strain IFM 10152) OX=247156 GN=trpS PE=3 SV=1
142.	tr|Q5Z264|Q5Z264_NOCFA	200	54276	22 (9)	10 (5)	Putative peptidase OS=Nocardia farcinica (strain IFM 10152) OX=247156 GN=NFA_6320 PE=4 SV=1
143.	tr|Q5Z0X1|Q5Z0X1_NOCFA	198	40125	17 (7)	8 (4)	Putative acyl-CoA thiolase OS=Nocardia farcinica (strain IFM 10152) OX=247156 GN=fadA4 PE=3 SV=1
144.	sp|Q5YRX6|SYE_NOCFA	196	54275	29 (8)	22 (6)	Glutamate--tRNA ligase OS=Nocardia farcinica (strain IFM 10152) OX=247156 GN=gltX PE=3 SV=1
145.	tr|Q5YN43|Q5YN43_NOCFA	190	46152	25 (9)	18 (5)	Putative transporter substrate-binding protein OS=Nocardia farcinica (strain IFM 10152) OX=247156 GN=NFA_55460 PE=4 SV=1
146.	tr|Q5YXT5|Q5YXT5_NOCFA	186	27230	20 (5)	11 (3)	Putative enoyl-CoA hydratase/isomerase family protein OS=Nocardia farcinica (strain IFM 10152) OX=247156 GN=echA11 PE=4 SV=1
147.	tr|Q5Z363|Q5Z363_NOCFA	186	76984	47 (4)	28 (3)	Putative 3-hydroxyacyl-CoA dehydrogenase OS=Nocardia farcinica (strain IFM 10152) OX=247156 GN=fadB PE=4 SV=1
148.	tr|Q5YXB1|Q5YXB1_NOCFA	184	17330	13 (6)	3 (3)	Uncharacterized protein OS=Nocardia farcinica (strain IFM 10152) OX=247156 GN=NFA_23330 PE=4 SV=1
149.	tr|Q5Z0Z6|Q5Z0Z6_NOCFA	182	37800	18 (7)	9 (3)	Threonine synthase OS=Nocardia farcinica (strain IFM 10152) OX=247156 GN=thrC PE=3 SV=1

No.	Accession	Score	Mass	Matches	Sequences	Name
150.	sp|Q5Z1K9|RPOA_NOCFA	176	38020	24 (8)	15 (5)	DNA-directed RNA polymerase subunit alpha OS=Nocardia farcinica (strain IFM 10152) OX=247156 GN=rpoA PE=3 SV=1
151.	tr|Q5YSX7|Q5YSX7_NOCFA	171	11454	12 (9)	2 (1)	Uncharacterized protein OS=Nocardia farcinica (strain IFM 10152) OX=247156 GN=NFA_38660 PE=4 SV=1
152.	tr|Q5YWH5|Q5YWH5_NOCFA	169	49206	14 (5)	9 (4)	Putative glycosyl hydrolase OS=Nocardia farcinica (strain IFM 10152) OX=247156 GN=NFA_26190 PE=3 SV=1
153.	sp|Q5YTQ6|TPIS_NOCFA	169	27569	26 (8)	10 (4)	Triosephosphate isomerase OS=Nocardia farcinica (strain IFM 10152) OX=247156 GN=tpiA PE=3 SV=1
154.	tr|Q5Z199|Q5Z199_NOCFA	168	66691	33 (4)	18 (2)	Succinate dehydrogenase flavoprotein subunit OS=Nocardia farcinica (strain IFM 10152) OX=247156 GN=sdhA PE=3 SV=1
155.	tr|Q5Z1M8|Q5Z1M8_NOCFA	168	41200	12 (6)	4 (2)	Uncharacterized protein OS=Nocardia farcinica (strain IFM 10152) OX=247156 GN=NFA_8180 PE=4 SV=1
156.	tr|Q5YPL3|Q5YPL3_NOCFA	165	28746	17 (6)	10 (3)	Putative bacteriocin family protein OS=Nocardia farcinica (strain IFM 10152) OX=247156 GN=NFA_50260 PE=4 SV=1
157.	tr|Q5Z288|Q5Z288_NOCFA	164	36613	16 (5)	13 (4)	Putative fatty acid desaturase OS=Nocardia farcinica (strain IFM 10152) OX=247156 GN=NFA_6080 PE=4 SV=1
158.	tr|Q5YYW5|Q5YYW5_NOCFA	163	19428	16 (8)	6 (3)	Uncharacterized protein OS=Nocardia farcinica (strain IFM 10152) OX=247156 GN=NFA_17800 PE=4 SV=1
159.	tr|Q5YNK7|Q5YNK7_NOCFA	159	36747	30 (10)	14 (6)	Putative fructose 1,6-bisphosphate aldolase OS=Nocardia farcinica (strain IFM 10152) OX=247156 GN=fba PE=3 SV=1
160.	tr|Q5YRZ5|Q5YRZ5_NOCFA	158	44397	8 (3)	5 (2)	Putative enoyl-CoA hydratase/isomerase family protein OS=Nocardia farcinica (strain IFM 10152) OX=247156 GN=NFA_41970 PE=3 SV=1

No.	Accession	Score	Mass	Matches	Sequences	Name
161.	tr|Q5YV76|Q5YV76_NOCFA	158	18749	11 (8)	6 (4)	Uncharacterized protein OS=Nocardia farcinica (strain IFM 10152) OX=247156 GN=NFA_30680 PE=4 SV=1
162.	tr|Q5Z2W6|Q5Z2W6_NOCFA	155	29222	10 (5)	7 (2)	Putative enoyl-CoA hydratase/isomerase family protein OS=Nocardia farcinica (strain IFM 10152) OX=247156 GN=echA1 PE=4 SV=1
163.	tr|Q5YSS5|Q5YSS5_NOCFA	154	32131	8 (5)	3 (2)	Uncharacterized protein OS=Nocardia farcinica (strain IFM 10152) OX=247156 GN=NFA_39180 PE=4 SV=1
164.	tr|Q5Z2D7|Q5Z2D7_NOCFA	154	51672	24 (7)	14 (5)	Adenylosuccinate lyase OS=Nocardia farcinica (strain IFM 10152) OX=247156 GN=purB PE=3 SV=1
165.	tr|Q5Z238|Q5Z238_NOCFA	153	48587	29 (10)	10 (6)	Citrate synthase OS=Nocardia farcinica (strain IFM 10152) OX=247156 GN=gltA PE=3 SV=1
166.	tr|Q5Z372|Q5Z372_NOCFA	152	35276	17 (6)	9 (3)	Uncharacterized protein OS=Nocardia farcinica (strain IFM 10152) OX=247156 GN=NFA_2770 PE=4 SV=1
167.	sp|Q5Z0Y1|ATPB_NOCFA	150	52379	26 (6)	20 (5)	ATP synthase subunit beta OS=Nocardia farcinica (strain IFM 10152) OX=247156 GN=atpD PE=3 SV=1
168.	tr|Q5YQE7|Q5YQE7_NOCFA	149	32672	14 (6)	7 (3)	2,3,4,5-tetrahydropyridine-2,6-dicarboxylate N-succinyltransferase OS=Nocardia farcinica (strain IFM 10152) OX=247156 GN=dapD PE=3 SV=1
169.	tr|Q5Z105|Q5Z105_NOCFA	146	28711	9 (5)	5 (3)	Putative enoyl-CoA hydratase/isomerase family protein OS=Nocardia farcinica (strain IFM 10152) OX=247156 GN=echA8 PE=3 SV=1
170.	tr|Q5YRP6|Q5YRP6_NOCFA	146	36622	17 (10)	8 (5)	Putative transporter OS=Nocardia farcinica (strain IFM 10152) OX=247156 GN=NFA_42960 PE=4 SV=1
171.	tr|Q5Z0M1|Q5Z0M1_NOCFA	145	51548	28 (12)	14 (4)	6-phosphogluconate dehydrogenase, decarboxylating OS=Nocardia farcinica (strain IFM 10152) OX=247156

No.	Accession	Score	Mass	Matches	Sequences	Name
						GN=gnd PE=3 SV=1
172.	tr|Q5YX07|Q5YX07_NOCFA	142	25910	31 (7)	10 (2)	Uncharacterized protein OS=Nocardia farcinica (strain IFM 10152) OX=247156 GN=NFA_24370 PE=4 SV=1
173.	tr|Q5YP11|Q5YP11_NOCFA	133	86325	22 (2)	15 (2)	5-methyltetrahydropteroyltriglutamate--homocysteine methyltransferase OS=Nocardia farcinica (strain IFM 10152) OX=247156 GN=metE PE=3 SV=1
174.	tr|Q5YR65|Q5YR65_NOCFA	130	28936	11 (5)	6 (3)	Uncharacterized protein OS=Nocardia farcinica (strain IFM 10152) OX=247156 GN=NFA_44750 PE=4 SV=1
175.	tr|Q5YZZ2|Q5YZZ2_NOCFA	130	36799	27 (6)	11 (5)	Putative transporter OS=Nocardia farcinica (strain IFM 10152) OX=247156 GN=NFA_14040 PE=4 SV=1
176.	tr|Q5YUT8|Q5YUT8_NOCFA	129	61675	10 (3)	9 (3)	Fumarate hydratase class I OS=Nocardia farcinica (strain IFM 10152) OX=247156 GN=fumA PE=3 SV=1
177.	tr|Q5YMF7|Q5YMF7_NOCFA	127	34040	12 (5)	7 (3)	Putative methyltransferase OS=Nocardia farcinica (strain IFM 10152) OX=247156 GN=PNF1_1090 PE=4 SV=1
178.	tr|Q5Z0J2|Q5Z0J2_NOCFA	127	54272	22 (6)	14 (3)	Putative aldehyde dehydrogenase OS=Nocardia farcinica (strain IFM 10152) OX=247156 GN=NFA_12040 PE=3 SV=1
179.	tr|Q5YPY3|Q5YPY3_NOCFA	126	58149	18 (2)	12 (1)	Methionine--tRNA ligase OS=Nocardia farcinica (strain IFM 10152) OX=247156 GN=metS PE=3 SV=1
180.	tr|Q5Z3M3|Q5Z3M3_NOCFA	126	32519	5 (2)	4 (1)	Uncharacterized protein OS=Nocardia farcinica (strain IFM 10152) OX=247156 GN=NFA_1260 PE=4 SV=1
181.	sp|Q5YWU1|MASZ_NOCFA	125	79381	35 (8)	13 (4)	Malate synthase G OS=Nocardia farcinica (strain IFM 10152) OX=247156 GN=glcB PE=3 SV=1
182.	tr|Q5Z239|Q5Z239_NOCFA	124	12666	8 (6)	4 (3)	Peptidyl-prolyl cis-trans isomerase OS=Nocardia farcinica (strain IFM 10152) OX=247156 GN=NFA_6570 PE=3 SV=1
183.	tr|Q5YR73|Q5YR73_NOCFA	122	28300	6 (3)	5 (3)	Uncharacterized protein OS=Nocardia farcinica (strain IFM

No.	Accession	Score	Mass	Matches	Sequences	Name
						10152) OX=247156 GN=NFA_44670 PE=4 SV=1
184.	tr|Q5YWY4|Q5YWY4_NOCFA	122	26313	7 (2)	4 (2)	Uncharacterized protein OS=Nocardia farcinica (strain IFM 10152) OX=247156 GN=NFA_24600 PE=4 SV=1
185.	tr|Q5Z0R9|Q5Z0R9_NOCFA	121	29593	19 (6)	10 (3)	Uncharacterized protein OS=Nocardia farcinica (strain IFM 10152) OX=247156 GN=NFA_11270 PE=4 SV=1
186.	tr|Q5YWG9|Q5YWG9_NOCFA	121	32485	15 (5)	8 (4)	Uncharacterized protein OS=Nocardia farcinica (strain IFM 10152) OX=247156 GN=NFA_26250 PE=4 SV=1
187.	tr|Q5Z0J0|Q5Z0J0_NOCFA	120	50152	14 (6)	8 (2)	Putative amidase OS=Nocardia farcinica (strain IFM 10152) OX=247156 GN=NFA_12060 PE=3 SV=1
188.	tr|Q5Z2D3|Q5Z2D3_NOCFA	119	79639	21 (7)	12 (5)	Putative protease OS=Nocardia farcinica (strain IFM 10152) OX=247156 GN=NFA_5630 PE=4 SV=1
189.	tr|Q5YW27|Q5YW27_NOCFA	119	36323	14 (4)	5 (2)	Uncharacterized protein OS=Nocardia farcinica (strain IFM 10152) OX=247156 GN=NFA_27670 PE=4 SV=1
190.	tr|Q5YVT9|Q5YVT9_NOCFA	118	31345	11 (3)	6 (2)	Putative fructose-1-phosphate kinase OS=Nocardia farcinica (strain IFM 10152) OX=247156 GN=fruK PE=3 SV=1
191.	sp|Q5YZ53|AMPA_NOCFA	115	51960	23 (5)	12 (3)	Probable cytosol aminopeptidase OS=Nocardia farcinica (strain IFM 10152) OX=247156 GN=pepA PE=3 SV=1
192.	tr|Q5YQ10|Q5YQ10_NOCFA	113	35704	18 (6)	11 (4)	Ribose-phosphate pyrophosphokinase OS=Nocardia farcinica (strain IFM 10152) OX=247156 GN=prsA PE=3 SV=1
193.	tr|Q5Z3G8|Q5Z3G8_NOCFA	113	37292	29 (5)	12 (3)	Putative mycolyltransferase OS=Nocardia farcinica (strain IFM 10152) OX=247156 GN=fbpA PE=4 SV=1
194.	tr|Q5YXW0|Q5YXW0_NOCFA	113	55762	19 (5)	10 (3)	Putative transporter substrate-binding protein OS=Nocardia farcinica (strain IFM 10152) OX=247156 GN=NFA_21350 PE=4 SV=1
195.	tr|Q5YS46|Q5YS46_NOCFA	112	28655	10 (3)	5 (2)	Signal peptidase I OS=Nocardia farcinica (strain IFM 10152)

No.	Accession	Score	Mass	Matches	Sequences	Name
						OX=247156 GN=NFA_41460 PE=3 SV=1
196.	tr|Q5Z184|Q5Z184_NOCFA	112	25523	11 (3)	6 (1)	Putative enoyl-CoA hydratase/isomerase family protein OS=Nocardia farcinica (strain IFM 10152) OX=247156 GN=NFA_9620 PE=4 SV=1
197.	sp|Q5YYD3|ASSY_NOCFA	111	44110	18 (3)	13 (3)	Argininosuccinate synthase OS=Nocardia farcinica (strain IFM 10152) OX=247156 GN=argG PE=3 SV=1
198.	tr|Q5YVP9|Q5YVP9_NOCFA	108	23084	13 (2)	7 (1)	Putative two-component system response regulator OS=Nocardia farcinica (strain IFM 10152) OX=247156 GN=NFA_28950 PE=4 SV=1
199.	tr|Q5YZD0|Q5YZD0_NOCFA	108	30795	13 (4)	7 (2)	Putative malonyl-CoA-ACP acyltransferase OS=Nocardia farcinica (strain IFM 10152) OX=247156 GN=fabD PE=4 SV=1
200.	tr|Q5YYM5|Q5YYM5_NOCFA	107	22144	17 (5)	9 (3)	Putative two-component system response regulator OS=Nocardia farcinica (strain IFM 10152) OX=247156 GN=NFA_18700 PE=4 SV=1
201.	tr|Q5YNM7|Q5YNM7_NOCFA	105	51089	21 (5)	12 (3)	Putative ferredoxin reductase OS=Nocardia farcinica (strain IFM 10152) OX=247156 GN=NFA_53620 PE=4 SV=1
202.	tr|Q5YTT5|Q5YTT5_NOCFA	105	28083	20 (3)	7 (2)	Putative ABC transporter ATP-binding protein OS=Nocardia farcinica (strain IFM 10152) OX=247156 GN=NFA_35580 PE=4 SV=1
203.	tr|Q5Z3Q2|Q5Z3Q2_NOCFA	103	53893	24 (3)	20 (3)	Putative glutamate synthase small subunit OS=Nocardia farcinica (strain IFM 10152) OX=247156 GN=gltD PE=4 SV=1
204.	sp|Q5YRT4|GATA_NOCFA	102	51640	17 (3)	14 (3)	Glutamyl-tRNA(Gln) amidotransferase subunit A OS=Nocardia farcinica (strain IFM 10152) OX=247156

No.	Accession	Score	Mass	Matches	Sequences	Name
						GN=gatA PE=3 SV=1
205.	tr|Q5YU26|Q5YU26_NOCFA	101	64791	28 (6)	17 (6)	Putative methylmalonyl-CoA mutase beta subunit OS=Nocardia farcinica (strain IFM 10152) OX=247156 GN=NFA_34670 PE=4 SV=1
206.	sp|Q5YN65|SYL_NOCFA	100	105730	30 (8)	24 (7)	Leucine--tRNA ligase OS=Nocardia farcinica (strain IFM 10152) OX=247156 GN=leuS PE=3 SV=1
207.	tr|Q5Z179|Q5Z179_NOCFA	100	49217	19 (3)	13 (3)	Putative dihydrolipoamide dehydrogenase OS=Nocardia farcinica (strain IFM 10152) OX=247156 GN=NFA_9670 PE=4 SV=1
208.	tr|Q5YS01|Q5YS01_NOCFA	100	121843	49 (7)	28 (5)	Pyruvate carboxylase OS=Nocardia farcinica (strain IFM 10152) OX=247156 GN=NFA_41910 PE=4 SV=1
209.	tr|Q5YMT0|Q5YMT0_NOCFA	99	131543	34 (3)	21 (2)	Putative membrane protein OS=Nocardia farcinica (strain IFM 10152) OX=247156 GN=NFA_56590 PE=4 SV=1
210.	sp|Q5YRN0|NADE_NOCFA	99	29640	14 (3)	9 (3)	NH(3)-dependent NAD(+) synthetase OS=Nocardia farcinica (strain IFM 10152) OX=247156 GN=nadE PE=3 SV=1
211.	sp|Q5YT53|AHPD_NOCFA	98	19088	16 (5)	9 (2)	Alkyl hydroperoxide reductase AhpD OS=Nocardia farcinica (strain IFM 10152) OX=247156 GN=ahpD PE=3 SV=1
212.	tr|Q5Z341|Q5Z341_NOCFA	96	66260	43 (7)	26 (6)	2-isopropylmalate synthase OS=Nocardia farcinica (strain IFM 10152) OX=247156 GN=leuA PE=3 SV=1
213.	tr|Q5YXR6|Q5YXR6_NOCFA	95	16181	5 (3)	3 (1)	Uncharacterized protein OS=Nocardia farcinica (strain IFM 10152) OX=247156 GN=NFA_21790 PE=4 SV=1
214.	tr|Q5YMM0|Q5YMM0_NOCFA	94	23903	8 (3)	5 (2)	Putative thioredoxin OS=Nocardia farcinica (strain IFM 10152) OX=247156 GN=PNF1_450 PE=4 SV=1
215.	tr|Q5Z3H3|Q5Z3H3_NOCFA	93	46881	33 (5)	21 (4)	Putative UDP-galactopyranose mutase OS=Nocardia farcinica (strain IFM 10152) OX=247156 GN=glf PE=4 SV=1

No.	Accession	Score	Mass	Matches	Sequences	Name
216.	sp|Q5Z1U0|AMIE_NOCFA	92	38672	24 (8)	9 (3)	Aliphatic amidase OS=Nocardia farcinica (strain IFM 10152) OX=247156 GN=amiE PE=3 SV=1
217.	tr|Q5YRR3|Q5YRR3_NOCFA	91	27466	14 (3)	8 (2)	Putative electron transfer flavoprotein beta subunit OS=Nocardia farcinica (strain IFM 10152) OX=247156 GN=NFA_42790 PE=4 SV=1
218.	tr|Q5YX81|Q5YX81_NOCFA	90	29061	8 (2)	5 (1)	Uncharacterized protein OS=Nocardia farcinica (strain IFM 10152) OX=247156 GN=NFA_23630 PE=4 SV=1
219.	tr|Q5Z157|Q5Z157_NOCFA	90	63432	26 (3)	19 (2)	Putative acyl-CoA carboxylase alpha subunit OS=Nocardia farcinica (strain IFM 10152) OX=247156 GN=NFA_9890 PE=4 SV=1
220.	sp|Q5Z048|SYV_NOCFA	90	98946	52 (6)	22 (4)	Valine--tRNA ligase OS=Nocardia farcinica (strain IFM 10152) OX=247156 GN=valS PE=3 SV=1
221.	tr|Q5Z331|Q5Z331_NOCFA	89	44994	12 (3)	6 (2)	Aspartokinase OS=Nocardia farcinica (strain IFM 10152) OX=247156 GN=NFA_3180 PE=3 SV=1
222.	tr|Q5YP71|Q5YP71_NOCFA	88	55119	15 (4)	8 (3)	Putative uroporphyrin-III C-methyltransferase/uroporphyrinogen-III synthase OS=Nocardia farcinica (strain IFM 10152) OX=247156 GN=hemDX PE=4 SV=1
223.	tr|Q5YXS3|Q5YXS3_NOCFA	88	26140	8 (3)	3 (2)	Uncharacterized protein OS=Nocardia farcinica (strain IFM 10152) OX=247156 GN=NFA_21720 PE=4 SV=1
224.	tr|Q5Z356|Q5Z356_NOCFA	87	20822	12 (5)	8 (5)	Uncharacterized protein OS=Nocardia farcinica (strain IFM 10152) OX=247156 GN=NFA_2930 PE=4 SV=1
225.	tr|Q5Z091|Q5Z091_NOCFA	86	62426	30 (4)	15 (3)	Putative ABC transporter ATP-binding protein OS=Nocardia farcinica (strain IFM 10152) OX=247156 GN=NFA_13050 PE=4 SV=1

No.	Accession	Score	Mass	Matches	Sequences	Name
226.	tr|Q5Z217|Q5Z217_NOCFA	86	21838	9 (3)	6 (2)	Uncharacterized protein OS=Nocardia farcinica (strain IFM 10152) OX=247156 GN=NFA_6790 PE=4 SV=1
227.	tr|Q5YVU3|Q5YVU3_NOCFA	86	23199	7 (2)	3 (1)	Uncharacterized protein OS=Nocardia farcinica (strain IFM 10152) OX=247156 GN=NFA_28510 PE=4 SV=1
228.	tr|Q5Z228|Q5Z228_NOCFA	85	14225	11 (3)	1 (1)	Uncharacterized protein OS=Nocardia farcinica (strain IFM 10152) OX=247156 GN=NFA_6680 PE=4 SV=1
229.	tr|Q5YSV8|Q5YSV8_NOCFA	85	14540	5 (2)	4 (2)	Uncharacterized protein OS=Nocardia farcinica (strain IFM 10152) OX=247156 GN=NFA_38850 PE=4 SV=1
230.	sp|Q5Z3N4|HTPG_NOCFA	85	74610	38 (6)	24 (4)	Chaperone protein HtpG OS=Nocardia farcinica (strain IFM 10152) OX=247156 GN=htpG PE=3 SV=1
231.	sp|Q5Z1F9|CH601_NOCFA	84	55769	15 (3)	13 (3)	60 kDa chaperonin 1 OS=Nocardia farcinica (strain IFM 10152) OX=247156 GN=groL1 PE=3 SV=1
232.	tr|Q5YN64|Q5YN64_NOCFA	84	29160	25 (3)	8 (2)	Putative polysaccharide deacetylase OS=Nocardia farcinica (strain IFM 10152) OX=247156 GN=NFA_55250 PE=4 SV=1
233.	tr|Q5YRV3|Q5YRV3_NOCFA	82	48280	21 (8)	14 (5)	Uncharacterized protein OS=Nocardia farcinica (strain IFM 10152) OX=247156 GN=NFA_42390 PE=4 SV=1
234.	tr|Q5Z107|Q5Z107_NOCFA	81	37698	10 (4)	7 (3)	Putative enoyl-CoA hydratase/isomerase family protein OS=Nocardia farcinica (strain IFM 10152) OX=247156 GN=NFA_10390 PE=4 SV=1
235.	tr|Q5YP45|Q5YP45_NOCFA	81	26059	5 (3)	4 (2)	Putative short chain dehydrogenase OS=Nocardia farcinica (strain IFM 10152) OX=247156 GN=NFA_51940 PE=3 SV=1
236.	tr|Q5YX84|Q5YX84_NOCFA	80	25956	11 (3)	9 (2)	Putative short chain dehydrogenase OS=Nocardia farcinica (strain IFM 10152) OX=247156 GN=NFA_23600 PE=4 SV=1
237.	tr|Q5Z339|Q5Z339_NOCFA	80	61022	21 (2)	12 (2)	Putative hydrolase OS=Nocardia farcinica (strain IFM 10152) OX=247156 GN=NFA_3100 PE=4 SV=1

No.	Accession	Score	Mass	Matches	Sequences	Name
238.	tr|Q5YQL4|Q5YQL4_NOCFA	80	23291	9 (2)	6 (1)	Uncharacterized protein OS=Nocardia farcinica (strain IFM 10152) OX=247156 GN=NFA_46760 PE=4 SV=1
239.	tr|Q5YQH4|Q5YQH4_NOCFA	79	33941	13 (4)	9 (3)	Putative lyase OS=Nocardia farcinica (strain IFM 10152) OX=247156 GN=NFA_47160 PE=3 SV=1
240.	sp|Q5Z062|CLPP3_NOCFA	79	24245	13 (2)	8 (2)	ATP-dependent Clp protease proteolytic subunit 3 OS=Nocardia farcinica (strain IFM 10152) OX=247156 GN=clpP3 PE=3 SV=1
241.	tr|Q5YPS8|Q5YPS8_NOCFA	78	20379	14 (1)	9 (1)	Putative molybdopterin biosynthesis protein OS=Nocardia farcinica (strain IFM 10152) OX=247156 GN=NFA_49610 PE=4 SV=1
242.	sp|Q5YUX2|PSB_NOCFA	76	30025	13 (3)	9 (3)	Proteasome subunit beta OS=Nocardia farcinica (strain IFM 10152) OX=247156 GN=prcB PE=3 SV=1
243.	tr|Q5YNB7|Q5YNB7_NOCFA	76	40969	11 (1)	11 (1)	Uncharacterized protein OS=Nocardia farcinica (strain IFM 10152) OX=247156 GN=NFA_54720 PE=4 SV=1
244.	tr|Q5Z3G6|Q5Z3G6_NOCFA	76	37328	23 (6)	14 (4)	Putative mycolyltransferase OS=Nocardia farcinica (strain IFM 10152) OX=247156 GN=fbpC PE=4 SV=1
245.	tr|Q5YT48|Q5YT48_NOCFA	75	43211	20 (2)	10 (1)	Putative aminotransferase OS=Nocardia farcinica (strain IFM 10152) OX=247156 GN=NFA_37950 PE=3 SV=1
246.	sp|Q5YTY9|KDGD_NOCFA	75	32397	13 (4)	8 (2)	Probable 5-dehydro-4-deoxyglucarate dehydratase OS=Nocardia farcinica (strain IFM 10152) OX=247156 GN=NFA_35040 PE=3 SV=1
247.	tr|Q5YPC1|Q5YPC1_NOCFA	74	38491	5 (3)	4 (2)	UPF0336 protein NFA_51180 OS=Nocardia farcinica (strain IFM 10152) OX=247156 GN=NFA_51180 PE=3 SV=1
248.	tr|Q5YPV8|Q5YPV8_NOCFA	74	27718	7 (2)	7 (2)	Putative lipoprotein OS=Nocardia farcinica (strain IFM 10152) OX=247156 GN=NFA_49310 PE=4 SV=1

No.	Accession	Score	Mass	Matches	Sequences	Name
249.	tr|Q5Z106|Q5Z106_NOCFA	73	30399	9 (1)	3 (1)	3-hydroxyisobutyrate dehydrogenase OS=Nocardia farcinica (strain IFM 10152) OX=247156 GN=NFA_10400 PE=3 SV=1
250.	tr|Q5YP43|Q5YP43_NOCFA	72	43067	2 (1)	2 (1)	Uncharacterized protein OS=Nocardia farcinica (strain IFM 10152) OX=247156 GN=NFA_51960 PE=4 SV=1
251.	sp|Q5Z0Y2|ATPG_NOCFA	72	35256	12 (2)	8 (2)	ATP synthase gamma chain OS=Nocardia farcinica (strain IFM 10152) OX=247156 GN=atpG PE=3 SV=1
252.	tr|Q5Z3N9|Q5Z3N9_NOCFA	70	17743	4 (1)	3 (1)	Uncharacterized protein OS=Nocardia farcinica (strain IFM 10152) OX=247156 GN=NFA_1100 PE=4 SV=1
253.	tr|Q5YTF8|Q5YTF8_NOCFA	69	43716	13 (5)	4 (2)	Putative flavohemoprotein OS=Nocardia farcinica (strain IFM 10152) OX=247156 GN=NFA_36850 PE=3 SV=1
254.	tr|Q5Z292|Q5Z292_NOCFA	68	24332	14 (2)	10 (1)	Phosphate-specific transport system accessory protein PhoU OS=Nocardia farcinica (strain IFM 10152) OX=247156 GN=phoU PE=3 SV=1
255.	tr|Q5Z3M8|Q5Z3M8_NOCFA	68	22980	15 (7)	3 (1)	Superoxide dismutase OS=Nocardia farcinica (strain IFM 10152) OX=247156 GN=sodF PE=3 SV=1
256.	tr|Q5YMD9|Q5YMD9_NOCFA	68	38672	11 (3)	9 (1)	Uncharacterized protein OS=Nocardia farcinica (strain IFM 10152) OX=247156 GN=PNF1_1270 PE=4 SV=1
257.	tr|Q5Z375|Q5Z375_NOCFA	68	46775	31 (4)	16 (4)	Uncharacterized protein OS=Nocardia farcinica (strain IFM 10152) OX=247156 GN=NFA_2740 PE=4 SV=1
258.	tr|Q5Z1A0|Q5Z1A0_NOCFA	67	29297	14 (1)	13 (1)	Putative succinate dehydrogenase iron-sulfur subunit OS=Nocardia farcinica (strain IFM 10152) OX=247156 GN=sdhB PE=4 SV=1
259.	sp|Q5YYJ6|COAE_NOCFA	66	39317	24 (1)	11 (1)	Dephospho-CoA kinase OS=Nocardia farcinica (strain IFM 10152) OX=247156 GN=coaE PE=3 SV=1
260.	tr|Q5YN60|Q5YN60_NOCFA	66	29805	12 (3)	8 (3)	Putative glycosyl hydrolase OS=Nocardia farcinica (strain

No.	Accession	Score	Mass	Matches	Sequences	Name
						IFM 10152) OX=247156 GN=NFA_55290 PE=4 SV=1
261.	tr|Q5YP51|Q5YP51_NOCFA	65	73378	35 (4)	18 (2)	Putative peptidase OS=Nocardia farcinica (strain IFM 10152) OX=247156 GN=NFA_51880 PE=4 SV=1
262.	tr|Q5YNU0|Q5YNU0_NOCFA	65	18279	11 (4)	5 (2)	Uncharacterized protein OS=Nocardia farcinica (strain IFM 10152) OX=247156 GN=NFA_52990 PE=4 SV=1
263.	tr|Q5YV18|Q5YV18_NOCFA	64	55361	14 (1)	9 (1)	Putative TldD protein OS=Nocardia farcinica (strain IFM 10152) OX=247156 GN=tldD PE=4 SV=1
264.	sp|Q5Z2A5|Y591_NOCFA	64	25163	9 (2)	7 (1)	UPF0678 fatty acid-binding protein-like protein NFA_5910 OS=Nocardia farcinica (strain IFM 10152) OX=247156 GN=NFA_5910 PE=3 SV=1
265.	tr|Q5Z1J3|Q5Z1J3_NOCFA	63	10522	11 (4)	4 (2)	ESAT-6-like protein OS=Nocardia farcinica (strain IFM 10152) OX=247156 GN=NFA_8530 PE=3 SV=1
266.	tr|Q5YST4|Q5YST4_NOCFA	62	39666	21 (5)	10 (3)	Uncharacterized protein OS=Nocardia farcinica (strain IFM 10152) OX=247156 GN=NFA_39090 PE=4 SV=1
267.	tr|Q5YXS8|Q5YXS8_NOCFA	61	14082	15 (3)	3 (2)	Uncharacterized protein OS=Nocardia farcinica (strain IFM 10152) OX=247156 GN=NFA_21670 PE=4 SV=1
268.	tr|Q5Z3K2|Q5Z3K2_NOCFA	61	57970	17 (1)	8 (1)	Uncharacterized protein OS=Nocardia farcinica (strain IFM 10152) OX=247156 GN=NFA_1470 PE=4 SV=1
269.	sp|Q5YYW9|SYI_NOCFA	61	116836	19 (4)	15 (3)	Isoleucine--tRNA ligase OS=Nocardia farcinica (strain IFM 10152) OX=247156 GN=ileS PE=3 SV=1
270.	tr|Q5YPQ3|Q5YPQ3_NOCFA	60	55129	21 (3)	9 (2)	Bifunctional purine biosynthesis protein PurH OS=Nocardia farcinica (strain IFM 10152) OX=247156 GN=purHJ PE=3 SV=1
271.	tr|Q5YT61|Q5YT61_NOCFA	60	24404	16 (2)	6 (2)	Uncharacterized protein OS=Nocardia farcinica (strain IFM 10152) OX=247156 GN=NFA_37820 PE=4 SV=1

No.	Accession	Score	Mass	Matches	Sequences	Name
272.	tr|Q5Z364|Q5Z364_NOCFA	60	42451	9 (3)	6 (2)	Putative acyl-CoA thiolase OS=Nocardia farcinica (strain IFM 10152) OX=247156 GN=fadA PE=3 SV=1
273.	sp|Q5YS62|EFTS_NOCFA	59	29395	20 (1)	11 (1)	Elongation factor Ts OS=Nocardia farcinica (strain IFM 10152) OX=247156 GN=tsf PE=3 SV=1
274.	tr|Q5YXM0|Q5YXM0_NOCFA	59	47018	31 (7)	13 (3)	Putative alcohol dehydrogenase OS=Nocardia farcinica (strain IFM 10152) OX=247156 GN=NFA_22240 PE=4 SV=1
275.	tr|Q5Z2T8|Q5Z2T8_NOCFA	59	54326	16 (3)	13 (2)	Lysine--tRNA ligase OS=Nocardia farcinica (strain IFM 10152) OX=247156 GN=lysS PE=3 SV=1
276.	tr|Q5Z0M5|Q5Z0M5_NOCFA	58	21875	13 (3)	2 (1)	ATP-dependent Clp protease proteolytic subunit OS=Nocardia farcinica (strain IFM 10152) OX=247156 GN=clp3 PE=3 SV=1
277.	tr|Q5YP46|Q5YP46_NOCFA	57	28977	8 (3)	4 (1)	Putative hydrolase OS=Nocardia farcinica (strain IFM 10152) OX=247156 GN=NFA_51930 PE=4 SV=1
278.	tr|Q5YY54|Q5YY54_NOCFA	57	11554	3 (2)	1 (1)	Uncharacterized protein OS=Nocardia farcinica (strain IFM 10152) OX=247156 GN=NFA_20410 PE=4 SV=1
279.	tr|Q5YNS2|Q5YNS2_NOCFA	57	28815	14 (3)	4 (2)	Uncharacterized protein OS=Nocardia farcinica (strain IFM 10152) OX=247156 GN=NFA_53170 PE=4 SV=1
280.	tr|Q5YNJ1|Q5YNJ1_NOCFA	56	20069	5 (1)	4 (1)	Orotate phosphoribosyltransferase OS=Nocardia farcinica (strain IFM 10152) OX=247156 GN=pyrE PE=3 SV=1
281.	tr|Q5YN92|Q5YN92_NOCFA	56	59510	22 (5)	13 (2)	Putative penicillin-binding protein OS=Nocardia farcinica (strain IFM 10152) OX=247156 GN=NFA_54970 PE=4 SV=1
282.	tr|Q5YVB3|Q5YVB3_NOCFA	56	44310	13 (3)	5 (1)	Uncharacterized protein OS=Nocardia farcinica (strain IFM 10152) OX=247156 GN=NFA_30310 PE=4 SV=1
283.	tr|Q5Z3Q1|Q5Z3Q1_NOCFA	56	31276	23 (1)	9 (1)	Uncharacterized protein OS=Nocardia farcinica (strain IFM 10152) OX=247156 GN=NFA_980 PE=4 SV=1

No.	Accession	Score	Mass	Matches	Sequences	Name
284.	sp|Q5YYD2|ARLY_NOCFA	56	49651	14 (2)	7 (2)	Argininosuccinate lyase OS=Nocardia farcinica (strain IFM 10152) OX=247156 GN=argH PE=3 SV=1
285.	sp|Q5Z1E9|GUAA_NOCFA	56	56527	15 (3)	9 (2)	GMP synthase [glutamine-hydrolyzing] OS=Nocardia farcinica (strain IFM 10152) OX=247156 GN=guaA PE=3 SV=1
286.	sp|Q5YNN4|FGD_NOCFA	56	36960	17 (2)	12 (2)	F420-dependent glucose-6-phosphate dehydrogenase OS=Nocardia farcinica (strain IFM 10152) OX=247156 GN=fgd PE=3 SV=1
287.	tr|Q5Z109|Q5Z109_NOCFA	56	54533	23 (3)	11 (3)	Putative methylmalonic acid semialdehyde dehydrogenase OS=Nocardia farcinica (strain IFM 10152) OX=247156 GN=NFA_10370 PE=4 SV=1
288.	tr|Q5YU17|Q5YU17_NOCFA	55	29339	7 (2)	6 (2)	Enoyl-[acyl-carrier-protein] reductase [NADH] OS=Nocardia farcinica (strain IFM 10152) OX=247156 GN=inhA PE=3 SV=1
289.	tr|Q5YYY5|Q5YYY5_NOCFA	55	66602	36 (3)	21 (2)	Putative penicillin-binding protein OS=Nocardia farcinica (strain IFM 10152) OX=247156 GN=NFA_17600 PE=4 SV=1
290.	sp|Q5YTN0|METK_NOCFA	55	43052	14 (3)	8 (3)	S-adenosylmethionine synthase OS=Nocardia farcinica (strain IFM 10152) OX=247156 GN=metK PE=3 SV=1
291.	tr|Q5Z305|Q5Z305_NOCFA	54	16161	5 (2)	3 (1)	Uncharacterized protein OS=Nocardia farcinica (strain IFM 10152) OX=247156 GN=NFA_3440 PE=4 SV=1
292.	tr|Q5Z2B7|Q5Z2B7_NOCFA	54	53589	17 (1)	6 (1)	Amidophosphoribosyltransferase OS=Nocardia farcinica (strain IFM 10152) OX=247156 GN=purF PE=3 SV=1
293.	tr|Q5Z1Y4|Q5Z1Y4_NOCFA	52	58401	16 (3)	10 (3)	Putative acyl-CoA synthetase OS=Nocardia farcinica (strain IFM 10152) OX=247156 GN=NFA_7120 PE=4 SV=1
294.	sp|Q5YTM7|KGUA_NOCFA	51	21294	4 (1)	3 (1)	Guanylate kinase OS=Nocardia farcinica (strain IFM 10152)

No.	Accession	Score	Mass	Matches	Sequences	Name
						OX=247156 GN=gmk PE=3 SV=1
295.	tr|Q5Z2Q3|Q5Z2Q3_NOCFA	51	93080	46 (2)	22 (2)	Putative trehalose-6-phosphate phophatase OS=Nocardia farcinica (strain IFM 10152) OX=247156 GN=NFA_4460 PE=4 SV=1
296.	tr|Q5Z2E1|Q5Z2E1_NOCFA	50	43205	11 (1)	7 (1)	Phosphoribosylamine--glycine ligase OS=Nocardia farcinica (strain IFM 10152) OX=247156 GN=purD PE=3 SV=1
297.	tr|Q5YYM4|Q5YYM4_NOCFA	50	37094	9 (1)	8 (1)	Putative transporter OS=Nocardia farcinica (strain IFM 10152) OX=247156 GN=NFA_18710 PE=4 SV=1
298.	tr|Q5YPA1|Q5YPA1_NOCFA	49	33413	6 (1)	6 (1)	1,4-dihydroxy-2-naphthoyl-CoA synthase OS=Nocardia farcinica (strain IFM 10152) OX=247156 GN=menB PE=3 SV=1
299.	sp|Q5YUV9|HIS1_NOCFA	49	30985	16 (4)	9 (3)	ATP phosphoribosyltransferase OS=Nocardia farcinica (strain IFM 10152) OX=247156 GN=hisG PE=3 SV=1
300.	sp|Q5YUX3|PSA_NOCFA	49	28146	8 (2)	7 (1)	Proteasome subunit alpha OS=Nocardia farcinica (strain IFM 10152) OX=247156 GN=prcA PE=3 SV=1
301.	tr|Q5YZ17|Q5YZ17_NOCFA	48	44039	7 (1)	4 (1)	Putative cytochrome c component OS=Nocardia farcinica (strain IFM 10152) OX=247156 GN=NFA_17280 PE=4 SV=1
302.	tr|Q5YX92|Q5YX92_NOCFA	48	15203	10 (1)	8 (1)	Uncharacterized protein OS=Nocardia farcinica (strain IFM 10152) OX=247156 GN=NFA_23520 PE=4 SV=1
303.	sp|Q5Z0Z9|SYR_NOCFA	48	59086	26 (2)	13 (2)	Arginine--tRNA ligase OS=Nocardia farcinica (strain IFM 10152) OX=247156 GN=argS PE=3 SV=1
304.	tr|Q5YU49|Q5YU49_NOCFA	47	31354	19 (2)	7 (2)	Uncharacterized protein OS=Nocardia farcinica (strain IFM 10152) OX=247156 GN=NFA_34440 PE=4 SV=1
305.	tr|Q5YQ50|Q5YQ50_NOCFA	47	16010	5 (2)	4 (1)	Uncharacterized protein OS=Nocardia farcinica (strain IFM 10152) OX=247156 GN=NFA_48390 PE=4 SV=1

No.	Accession	Score	Mass	Matches	Sequences	Name
306.	sp|Q5YRV8|ILVD3_NOCFA	47	64803	16 (3)	12 (3)	Dihydroxy-acid dehydratase 3 OS=Nocardia farcinica (strain IFM 10152) OX=247156 GN=ilvD3 PE=3 SV=1
307.	tr|Q5YW21|Q5YW21_NOCFA	47	51930	24 (3)	13 (2)	Putative succinate-semialdehyde dehydrogenase OS=Nocardia farcinica (strain IFM 10152) OX=247156 GN=gabD PE=3 SV=1
308.	tr|Q5Z0T5|Q5Z0T5_NOCFA	46	24245	12 (4)	4 (1)	Uncharacterized protein OS=Nocardia farcinica (strain IFM 10152) OX=247156 GN=NFA_11110 PE=4 SV=1
309.	tr|Q5YQK1|Q5YQK1_NOCFA	46	27812	6 (3)	5 (2)	Putative enoyl-CoA hydratase/isomerase family protein OS=Nocardia farcinica (strain IFM 10152) OX=247156 GN=echA19 PE=4 SV=1
310.	tr|Q5YZ13|Q5YZ13_NOCFA	46	51322	16 (2)	13 (2)	Phospho-2-dehydro-3-deoxyheptonate aldolase OS=Nocardia farcinica (strain IFM 10152) OX=247156 GN=aroG2 PE=3 SV=1
311.	tr|Q5YVK2|Q5YVK2_NOCFA	46	27289	3 (2)	1 (1)	Putative enoyl-CoA hydratase/isomerase family protein OS=Nocardia farcinica (strain IFM 10152) OX=247156 GN=echA13 PE=3 SV=1
312.	tr|Q5YTT9|Q5YTT9_NOCFA	45	14984	4 (1)	3 (1)	Uncharacterized protein OS=Nocardia farcinica (strain IFM 10152) OX=247156 GN=NFA_35540 PE=4 SV=1
313.	sp|Q5YPC5|RL1_NOCFA	44	25046	14 (1)	10 (1)	50S ribosomal protein L1 OS=Nocardia farcinica (strain IFM 10152) OX=247156 GN=rplA PE=3 SV=1
314.	tr|Q5Z3K1|Q5Z3K1_NOCFA	44	31610	1 (1)	1 (1)	Uncharacterized protein OS=Nocardia farcinica (strain IFM 10152) OX=247156 GN=NFA_1480 PE=4 SV=1
315.	sp|Q5YTK8|AROC_NOCFA	44	42329	14 (1)	10 (1)	Chorismate synthase OS=Nocardia farcinica (strain IFM 10152) OX=247156 GN=aroC PE=3 SV=1
316.	tr|Q5YQV1|Q5YQV1_NOCFA	43	45882	4 (1)	4 (1)	3-phosphoshikimate 1-carboxyvinyltransferase OS=Nocardia

No.	Accession	Score	Mass	Matches	Sequences	Name
						farcinica (strain IFM 10152) OX=247156 GN=aroA PE=3 SV=1
317.	tr|Q5Z0J9|Q5Z0J9_NOCFA	43	39732	10 (1)	6 (1)	Putative dehydrogenase OS=Nocardia farcinica (strain IFM 10152) OX=247156 GN=NFA_11970 PE=3 SV=1
318.	tr|Q5Z2E3|Q5Z2E3_NOCFA	43	15198	4 (2)	3 (2)	Uncharacterized protein OS=Nocardia farcinica (strain IFM 10152) OX=247156 GN=NFA_5530 PE=4 SV=1
319.	tr|Q5YUY5|Q5YUY5_NOCFA	43	39912	7 (2)	6 (2)	Putative peptidase OS=Nocardia farcinica (strain IFM 10152) OX=247156 GN=NFA_31590 PE=3 SV=1
320.	tr|Q5Z330|Q5Z330_NOCFA	42	36199	20 (1)	9 (1)	Aspartate-semialdehyde dehydrogenase OS=Nocardia farcinica (strain IFM 10152) OX=247156 GN=asd PE=3 SV=1
321.	tr|Q5YW18|Q5YW18_NOCFA	41	43422	6 (1)	3 (1)	Putative 3,4-dihydroxyphenylacetate 2,3-dioxygenase OS=Nocardia farcinica (strain IFM 10152) OX=247156 GN=NFA_27760 PE=3 SV=1
322.	sp|Q5YNP4|ACKA_NOCFA	41	42426	9 (1)	6 (1)	Acetate kinase OS=Nocardia farcinica (strain IFM 10152) OX=247156 GN=ackA PE=3 SV=1
323.	tr|Q5YW17|Q5YW17_NOCFA	41	54401	10 (2)	6 (2)	Putative 5-carboxymethyl-2-hydroxymuconate semialdehyde dehydrogenase OS=Nocardia farcinica (strain IFM 10152) OX=247156 GN=NFA_27770 PE=3 SV=1
324.	tr|Q5Z1M4|Q5Z1M4_NOCFA	41	10548	10 (2)	6 (2)	ESAT-6-like protein OS=Nocardia farcinica (strain IFM 10152) OX=247156 GN=NFA_8220 PE=3 SV=1
325.	tr|Q5YN29|Q5YN29_NOCFA	41	46733	15 (2)	11 (2)	Putative transporter OS=Nocardia farcinica (strain IFM 10152) OX=247156 GN=NFA_55600 PE=4 SV=1
326.	tr|Q5YMP3|Q5YMP3_NOCFA	41	55519	19 (4)	13 (2)	Putative multicopper oxidase OS=Nocardia farcinica (strain IFM 10152) OX=247156 GN=PNF1_230 PE=4 SV=1
327.	tr|Q5YTP6|Q5YTP6_NOCFA	41	65075	15 (1)	9 (1)	Putative gamma-glutamyltranspeptidase OS=Nocardia

No.	Accession	Score	Mass	Matches	Sequences	Name
						farcinica (strain IFM 10152) OX=247156 GN=ggt PE=4 SV=1
328.	tr|Q5YQI2|Q5YQI2_NOCFA	41	30033	14 (1)	7 (1)	Putative transporter substrate-binding protein OS=Nocardia farcinica (strain IFM 10152) OX=247156 GN=NFA_47080 PE=4 SV=1
329.	tr|Q5Z164|Q5Z164_NOCFA	40	54038	11 (2)	7 (2)	Putative aldehyde dehydrogenase OS=Nocardia farcinica (strain IFM 10152) OX=247156 GN=NFA_9820 PE=4 SV=1
330.	tr|Q5YQ89|Q5YQ89_NOCFA	40	36393	19 (1)	17 (1)	Fructose-1,6-bisphosphatase OS=Nocardia farcinica (strain IFM 10152) OX=247156 GN=NFA_48000 PE=3 SV=1
331.	tr|Q5Z3Q3|Q5Z3Q3_NOCFA	40	169224	28 (1)	24 (1)	Putative glutamate synthase large subunit OS=Nocardia farcinica (strain IFM 10152) OX=247156 GN=gltB PE=4 SV=1
332.	tr|Q5YTP2|Q5YTP2_NOCFA	40	21821	3 (1)	3 (1)	Putative riboflavin synthase alpha subunit OS=Nocardia farcinica (strain IFM 10152) OX=247156 GN=ribE PE=4 SV=1
333.	tr|Q5YQQ0|Q5YQQ0_NOCFA	40	24444	5 (1)	3 (1)	Uncharacterized protein OS=Nocardia farcinica (strain IFM 10152) OX=247156 GN=NFA_46400 PE=4 SV=1
334.	tr|Q5YST0|Q5YST0_NOCFA	40	193036	85 (4)	51 (2)	Putative phage tail OS=Nocardia farcinica (strain IFM 10152) OX=247156 GN=NFA_39130 PE=4 SV=1
335.	tr|Q5YM21|Q5YM21_NOCFA	40	22076	18 (1)	5 (1)	Uncharacterized protein OS=Nocardia farcinica (strain IFM 10152) OX=247156 GN=PNF2_820 PE=4 SV=1
336.	tr|Q5YN32|Q5YN32_NOCFA	39	84547	36 (2)	19 (2)	Putative penicillin-binding protein OS=Nocardia farcinica (strain IFM 10152) OX=247156 GN=NFA_55570 PE=4 SV=1
337.	tr|Q5YQ90|Q5YQ90_NOCFA	39	20291	9 (1)	5 (1)	Uncharacterized protein OS=Nocardia farcinica (strain IFM 10152) OX=247156 GN=NFA_47990 PE=4 SV=1
338.	tr|Q5YR96|Q5YR96_NOCFA	39	103176	32 (1)	25 (1)	Uncharacterized protein OS=Nocardia farcinica (strain IFM

No.	Accession	Score	Mass	Matches	Sequences	Name
						10152) OX=247156 GN=NFA_44440 PE=4 SV=1
339.	sp|Q5Z025|PROA_NOCFA	39	44108	11 (1)	9 (1)	Gamma-glutamyl phosphate reductase OS=Nocardia farcinica (strain IFM 10152) OX=247156 GN=proA PE=3 SV=1
340.	tr|Q5YQ88|Q5YQ88_NOCFA	38	49695	29 (5)	15 (3)	Fumarate hydratase class II OS=Nocardia farcinica (strain IFM 10152) OX=247156 GN=fumC PE=3 SV=1
341.	tr|Q5Z2R8|Q5Z2R8_NOCFA	38	20296	6 (1)	6 (1)	Uncharacterized protein OS=Nocardia farcinica (strain IFM 10152) OX=247156 GN=NFA_4310 PE=4 SV=1
342.	sp|Q5YRY1|LEUD_NOCFA	38	22512	11 (1)	8 (1)	3-isopropylmalate dehydratase small subunit OS=Nocardia farcinica (strain IFM 10152) OX=247156 GN=leuD PE=3 SV=1
343.	tr|Q5YQS9|Q5YQS9_NOCFA	38	24870	8 (1)	7 (1)	Putative two-component system response regulator OS=Nocardia farcinica (strain IFM 10152) OX=247156 GN=NFA_46110 PE=4 SV=1
344.	tr|Q5YR11|Q5YR11_NOCFA	37	49483	8 (1)	6 (1)	Uncharacterized protein OS=Nocardia farcinica (strain IFM 10152) OX=247156 GN=NFA_45290 PE=4 SV=1
345.	tr|Q5YU43|Q5YU43_NOCFA	37	33119	7 (1)	6 (1)	Putative short chain dehydrogenase OS=Nocardia farcinica (strain IFM 10152) OX=247156 GN=NFA_34500 PE=3 SV=1
346.	tr|Q5YRP9|Q5YRP9_NOCFA	37	43605	6 (1)	4 (1)	Putative phosphoserine phosphatase OS=Nocardia farcinica (strain IFM 10152) OX=247156 GN=serB PE=4 SV=1
347.	tr|Q5YU12|Q5YU12_NOCFA	36	49878	18 (3)	9 (2)	Putative invasin OS=Nocardia farcinica (strain IFM 10152) OX=247156 GN=inv PE=4 SV=1
348.	tr|Q5YZ55|Q5YZ55_NOCFA	36	60364	16 (2)	13 (1)	Dihydrolipoamide acetyltransferase component of pyruvate dehydrogenase complex OS=Nocardia farcinica (strain IFM 10152) OX=247156 GN=sucB PE=3 SV=1
349.	tr|Q5YVM6|Q5YVM6_NOCFA	36	35500	5 (2)	5 (2)	Putative lipoprotein OS=Nocardia farcinica (strain IFM 10152)

No.	Accession	Score	Mass	Matches	Sequences	Name
						OX=247156 GN=NFA_29180 PE=4 SV=1
350.	sp|Q5YYA4|SYY_NOCFA	36	46904	15 (1)	10 (1)	Tyrosine--tRNA ligase OS=Nocardia farcinica (strain IFM 10152) OX=247156 GN=tyrS PE=3 SV=1
351.	tr|Q5YPP2|Q5YPP2_NOCFA	36	51446	12 (2)	10 (2)	Putative aldehyde dehydrogenase OS=Nocardia farcinica (strain IFM 10152) OX=247156 GN=NFA_49970 PE=3 SV=1
352.	tr|Q5Z322|Q5Z322_NOCFA	35	31638	8 (1)	5 (1)	Putative oxidoreductase OS=Nocardia farcinica (strain IFM 10152) OX=247156 GN=NFA_3270 PE=4 SV=1
353.	tr|Q5Z373|Q5Z373_NOCFA	35	29919	12 (2)	6 (1)	Uncharacterized protein OS=Nocardia farcinica (strain IFM 10152) OX=247156 GN=NFA_2760 PE=4 SV=1
354.	tr|Q5YXG4|Q5YXG4_NOCFA	35	77376	13 (1)	12 (1)	Putative peptidase OS=Nocardia farcinica (strain IFM 10152) OX=247156 GN=NFA_22800 PE=4 SV=1
355.	tr|Q5YXX2|Q5YXX2_NOCFA	35	41387	9 (1)	8 (1)	Putative ABC transporter OS=Nocardia farcinica (strain IFM 10152) OX=247156 GN=NFA_21230 PE=4 SV=1
356.	tr|Q5YT91|Q5YT91_NOCFA	35	32075	13 (1)	8 (1)	Uncharacterized protein OS=Nocardia farcinica (strain IFM 10152) OX=247156 GN=NFA_37520 PE=4 SV=1
357.	sp|Q5Z2U3|PANC_NOCFA	35	33541	14 (1)	9 (1)	Pantothenate synthetase OS=Nocardia farcinica (strain IFM 10152) OX=247156 GN=panC PE=3 SV=1
358.	tr|Q5YQU8|Q5YQU8_NOCFA	35	50349	37 (1)	17 (1)	Glutamate dehydrogenase OS=Nocardia farcinica (strain IFM 10152) OX=247156 GN=NFA_45920 PE=3 SV=1
359.	tr|Q5YS25|Q5YS25_NOCFA	34	130192	65 (1)	40 (1)	Chromosome partition protein Smc OS=Nocardia farcinica (strain IFM 10152) OX=247156 GN=smc PE=3 SV=1
360.	sp|Q5Z0G2|HUTI_NOCFA	34	41647	10 (2)	6 (1)	Imidazolonepropionase OS=Nocardia farcinica (strain IFM 10152) OX=247156 GN=hutI PE=3 SV=1
361.	sp|Q5YPC8|RL7_NOCFA	33	13129	2 (1)	2 (1)	50S ribosomal protein L7/L12 OS=Nocardia farcinica (strain IFM 10152) OX=247156 GN=rplL PE=3 SV=1

No.	Accession	Score	Mass	Matches	Sequences	Name
362.	tr|Q5Z3F8|Q5Z3F8_NOCFA	33	17544	14 (1)	7 (1)	Peptidyl-prolyl cis-trans isomerase OS=Nocardia farcinica (strain IFM 10152) OX=247156 GN=NFA_1910 PE=3 SV=1
363.	tr|Q5YQQ4|Q5YQQ4_NOCFA	33	37643	4 (1)	4 (1)	Putative mannose-1-phosphate guanylyltransferase OS=Nocardia farcinica (strain IFM 10152) OX=247156 GN=NFA_46360 PE=4 SV=1
364.	tr|Q5YYJ0|Q5YYJ0_NOCFA	33	15843	17 (1)	8 (1)	Uncharacterized protein OS=Nocardia farcinica (strain IFM 10152) OX=247156 GN=NFA_19050 PE=4 SV=1
365.	tr|Q5YQ62|Q5YQ62_NOCFA	33	40080	8 (1)	7 (1)	Putative cystathionine gamma-synthase OS=Nocardia farcinica (strain IFM 10152) OX=247156 GN=metB PE=3 SV=1
366.	tr|Q5YW09|Q5YW09_NOCFA	33	55288	25 (1)	13 (1)	Putative oxidoreductase OS=Nocardia farcinica (strain IFM 10152) OX=247156 GN=NFA_27850 PE=4 SV=1
367.	tr|Q5YRN6|Q5YRN6_NOCFA	33	36661	10 (1)	8 (1)	Uncharacterized protein OS=Nocardia farcinica (strain IFM 10152) OX=247156 GN=NFA_43060 PE=4 SV=1
368.	tr|Q5YWT0|Q5YWT0_NOCFA	33	50106	13 (1)	12 (1)	Putative inosine-5'-monophosphate dehydrogenase OS=Nocardia farcinica (strain IFM 10152) OX=247156 GN=guaB2 PE=4 SV=1
369.	tr|Q5YUN5|Q5YUN5_NOCFA	32	42623	7 (2)	3 (2)	Putative lipase OS=Nocardia farcinica (strain IFM 10152) OX=247156 GN=NFA_32590 PE=4 SV=1
370.	sp|Q5YTA5|DCUP_NOCFA	32	37637	9 (1)	5 (1)	Uroporphyrinogen decarboxylase OS=Nocardia farcinica (strain IFM 10152) OX=247156 GN=hemE PE=3 SV=1
371.	tr|Q5YQI6|Q5YQI6_NOCFA	32	63971	10 (2)	7 (2)	Putative acyl-CoA synthetase OS=Nocardia farcinica (strain IFM 10152) OX=247156 GN=NFA_47040 PE=4 SV=1
372.	tr|Q5YV17|Q5YV17_NOCFA	32	49662	11 (1)	8 (1)	Uncharacterized protein OS=Nocardia farcinica (strain IFM 10152) OX=247156 GN=NFA_31270 PE=4 SV=1
373.	sp|Q5YQ76|GLYA_NOCFA	32	46089	24 (2)	15 (2)	Serine hydroxymethyltransferase OS=Nocardia farcinica

No.	Accession	Score	Mass	Matches	Sequences	Name
						(strain IFM 10152) OX=247156 GN=glyA PE=3 SV=1
374.	tr|Q5YN95|Q5YN95_NOCFA	32	55612	36 (1)	11 (1)	Uncharacterized protein OS=Nocardia farcinica (strain IFM 10152) OX=247156 GN=NFA_54940 PE=4 SV=1
375.	tr|Q5YSV3|Q5YSV3_NOCFA	31	34669	15 (1)	10 (1)	Riboflavin biosynthesis protein OS=Nocardia farcinica (strain IFM 10152) OX=247156 GN=ribF PE=3 SV=1
376.	tr|Q5YMJ2|Q5YMJ2_NOCFA	31	50721	6 (2)	3 (1)	Uncharacterized protein OS=Nocardia farcinica (strain IFM 10152) OX=247156 GN=PNF1_740 PE=4 SV=1
377.	tr|Q5YQR5|Q5YQR5_NOCFA	31	48593	16 (2)	9 (2)	Putative phosphomannomutase OS=Nocardia farcinica (strain IFM 10152) OX=247156 GN=cpsG PE=4 SV=1
378.	sp|Q5YRV4|GATB_NOCFA	31	54671	15 (2)	14 (2)	Aspartyl/glutamyl-tRNA(Asn/Gln) amidotransferase subunit B OS=Nocardia farcinica (strain IFM 10152) OX=247156 GN=gatB PE=3 SV=1
379.	sp|Q5Z0V1|RNPH_NOCFA	31	27287	5 (1)	4 (1)	Ribonuclease PH OS=Nocardia farcinica (strain IFM 10152) OX=247156 GN=rph PE=3 SV=1
380.	tr|Q5YZF1|Q5YZF1_NOCFA	31	45556	18 (1)	11 (1)	Uncharacterized protein OS=Nocardia farcinica (strain IFM 10152) OX=247156 GN=NFA_15940 PE=4 SV=1
381.	tr|Q5YMN8|Q5YMN8_NOCFA	30	86868	25 (2)	12 (2)	Putative cation-transporting ATPase OS=Nocardia farcinica (strain IFM 10152) OX=247156 GN=PNF1_280 PE=3 SV=1
382.	tr|Q5YRE3|Q5YRE3_NOCFA	30	54219	15 (3)	9 (1)	Putative phytoene desaturase OS=Nocardia farcinica (strain IFM 10152) OX=247156 GN=crtI2 PE=3 SV=1
383.	sp|Q5YVJ4|KATG_NOCFA	30	80217	23 (2)	14 (2)	Catalase-peroxidase OS=Nocardia farcinica (strain IFM 10152) OX=247156 GN=katG PE=3 SV=1
384.	tr|Q5YS42|Q5YS42_NOCFA	30	61057	21 (1)	13 (1)	Putative acyl-CoA synthetase OS=Nocardia farcinica (strain IFM 10152) OX=247156 GN=NFA_41500 PE=4 SV=1
385.	sp|Q5Z1I0|RL13_NOCFA	30	15937	14 (2)	8 (2)	50S ribosomal protein L13 OS=Nocardia farcinica (strain IFM

No.	Accession	Score	Mass	Matches	Sequences	Name
						10152) OX=247156 GN=rplM PE=3 SV=1
386.	tr|Q5YT57|Q5YT57_NOCFA	30	44857	12 (1)	8 (1)	Polyamine aminopropyltransferase OS=Nocardia farcinica (strain IFM 10152) OX=247156 GN=speE PE=3 SV=1
387.	tr|Q5YR12|Q5YR12_NOCFA	30	34673	8 (2)	8 (2)	Endopeptidase La OS=Nocardia farcinica (strain IFM 10152) OX=247156 GN=NFA_45280 PE=3 SV=1
388.	tr|Q5YQM1|Q5YQM1_NOCFA	30	27795	11 (1)	8 (1)	Putative endonuclease IV OS=Nocardia farcinica (strain IFM 10152) OX=247156 GN=NFA_46690 PE=4 SV=1
389.	tr|Q5YX96|Q5YX96_NOCFA	29	42642	19 (1)	6 (1)	Putative monooxygenase OS=Nocardia farcinica (strain IFM 10152) OX=247156 GN=NFA_23480 PE=4 SV=1
390.	tr|Q5YVL5|Q5YVL5_NOCFA	29	33313	9 (1)	6 (1)	Putative oxidoreductase OS=Nocardia farcinica (strain IFM 10152) OX=247156 GN=NFA_29290 PE=4 SV=1
391.	tr|Q5YU27|Q5YU27_NOCFA	29	82356	24 (1)	19 (1)	Putative methylmalonyl-CoA mutase alpha subunit OS=Nocardia farcinica (strain IFM 10152) OX=247156 GN=NFA_34660 PE=4 SV=1
392.	tr|Q5YTN3|Q5YTN3_NOCFA	29	36537	5 (2)	3 (1)	Putative lipase OS=Nocardia farcinica (strain IFM 10152) OX=247156 GN=NFA_36100 PE=4 SV=1
393.	tr|Q5Z1K2|Q5Z1K2_NOCFA	29	29222	4 (1)	3 (1)	S-methyl-5'-thioadenosine phosphorylase OS=Nocardia farcinica (strain IFM 10152) OX=247156 GN=mtnP PE=3 SV=1
394.	tr|Q5Z3K3|Q5Z3K3_NOCFA	29	32581	13 (1)	9 (1)	Putative transcriptional regulator OS=Nocardia farcinica (strain IFM 10152) OX=247156 GN=NFA_1460 PE=3 SV=1
395.	tr|Q5Z0U4|Q5Z0U4_NOCFA	29	24163	10 (1)	6 (1)	Putative transcriptional regulator OS=Nocardia farcinica (strain IFM 10152) OX=247156 GN=NFA_11020 PE=4 SV=1
396.	tr|Q5YPF5|Q5YPF5_NOCFA	29	33668	9 (2)	5 (2)	Putative dehydrogenase OS=Nocardia farcinica (strain IFM 10152) OX=247156 GN=NFA_50840 PE=4 SV=1

No.	Accession	Score	Mass	Matches	Sequences	Name
397.	tr|Q5Z2U4|Q5Z2U4_NOCFA	29	29831	4 (1)	4 (1)	Uncharacterized protein OS=Nocardia farcinica (strain IFM 10152) OX=247156 GN=NFA_4050 PE=4 SV=1
398.	sp|Q5YZ21|TRPD_NOCFA	29	35078	16 (1)	8 (1)	Anthranilate phosphoribosyltransferase OS=Nocardia farcinica (strain IFM 10152) OX=247156 GN=trpD PE=3 SV=1
399.	sp|Q5YQU1|SECA_NOCFA	28	104245	41 (1)	25 (1)	Protein translocase subunit SecA OS=Nocardia farcinica (strain IFM 10152) OX=247156 GN=secA PE=3 SV=1
400.	tr|Q5YXI1|Q5YXI1_NOCFA	28	23228	14 (1)	8 (1)	Putative transcriptional regulator OS=Nocardia farcinica (strain IFM 10152) OX=247156 GN=NFA_22630 PE=4 SV=1
401.	tr|Q5YMP8|Q5YMP8_NOCFA	28	33881	15 (2)	6 (1)	Putative ATP-binding protein OS=Nocardia farcinica (strain IFM 10152) OX=247156 GN=PNF1_180 PE=4 SV=1
402.	sp|Q5YNP9|THIE_NOCFA	28	24709	23 (1)	4 (1)	Thiamine-phosphate synthase OS=Nocardia farcinica (strain IFM 10152) OX=247156 GN=thiE PE=3 SV=1
403.	sp|Q5YQ11|GLMU_NOCFA	28	51744	15 (1)	12 (1)	Bifunctional protein GlmU OS=Nocardia farcinica (strain IFM 10152) OX=247156 GN=glmU PE=3 SV=1
404.	tr|Q5YUR6|Q5YUR6_NOCFA	28	36715	7 (1)	6 (1)	Uncharacterized protein OS=Nocardia farcinica (strain IFM 10152) OX=247156 GN=NFA_32280 PE=4 SV=1
405.	sp|Q5Z1H9|RS9_NOCFA	27	18567	5 (2)	3 (1)	30S ribosomal protein S9 OS=Nocardia farcinica (strain IFM 10152) OX=247156 GN=rpsI PE=3 SV=1
406.	tr|Q5YPC3|Q5YPC3_NOCFA	27	29371	10 (2)	5 (1)	Transcription termination/antitermination protein NusG OS=Nocardia farcinica (strain IFM 10152) OX=247156 GN=nusG PE=3 SV=1
407.	tr|Q5YP24|Q5YP24_NOCFA	27	21704	7 (1)	6 (1)	Uncharacterized protein OS=Nocardia farcinica (strain IFM 10152) OX=247156 GN=NFA_52150 PE=4 SV=1
408.	tr|Q5YXN3|Q5YXN3_NOCFA	27	37951	19 (2)	9 (1)	Putative 3-hydroxy-3-methylglutaryl-CoA reductase OS=Nocardia farcinica (strain IFM 10152) OX=247156

No.	Accession	Score	Mass	Matches	Sequences	Name
						GN=NFA_22110 PE=4 SV=1
409.	tr|Q5Z068|Q5Z068_NOCFA	27	29655	7 (2)	5 (1)	Putative formamidopyrimidine-DNA glycosylase OS=Nocardia farcinica (strain IFM 10152) OX=247156 GN=NFA_13280 PE=3 SV=1
410.	tr|Q5Z3I6|Q5Z3I6_NOCFA	27	28092	25 (1)	5 (1)	Putative transcriptional regulator OS=Nocardia farcinica (strain IFM 10152) OX=247156 GN=NFA_1630 PE=4 SV=1
411.	tr|Q5YTG8|Q5YTG8_NOCFA	27	29670	13 (1)	9 (1)	Uncharacterized protein OS=Nocardia farcinica (strain IFM 10152) OX=247156 GN=NFA_36750 PE=4 SV=1
412.	tr|Q5YR91|Q5YR91_NOCFA	26	24443	26 (1)	8 (1)	Uncharacterized protein OS=Nocardia farcinica (strain IFM 10152) OX=247156 GN=NFA_44490 PE=4 SV=1
413.	tr|Q5YME5|Q5YME5_NOCFA	26	23246	6 (1)	5 (1)	Orotidine 5'-phosphate decarboxylase OS=Nocardia farcinica (strain IFM 10152) OX=247156 GN=PNF1_1210 PE=3 SV=1
414.	tr|Q5YPB1|Q5YPB1_NOCFA	26	40614	4 (1)	3 (1)	Putative glycosyltransferase OS=Nocardia farcinica (strain IFM 10152) OX=247156 GN=NFA_51280 PE=4 SV=1
415.	tr|Q5Z0H3|Q5Z0H3_NOCFA	26	38289	9 (1)	6 (1)	Putative dehydrogenase OS=Nocardia farcinica (strain IFM 10152) OX=247156 GN=NFA_12230 PE=3 SV=1
416.	tr|Q5YVN3|Q5YVN3_NOCFA	26	18429	1 (1)	1 (1)	Uncharacterized protein OS=Nocardia farcinica (strain IFM 10152) OX=247156 GN=NFA_29110 PE=4 SV=1
417.	tr|Q5Z3K7|Q5Z3K7_NOCFA	26	46373	13 (1)	3 (1)	Uncharacterized protein OS=Nocardia farcinica (strain IFM 10152) OX=247156 GN=NFA_1420 PE=4 SV=1
418.	tr|Q5YWK1|Q5YWK1_NOCFA	26	64104	11 (1)	10 (1)	Putative oxidoreductase OS=Nocardia farcinica (strain IFM 10152) OX=247156 GN=NFA_25930 PE=4 SV=1
419.	tr|Q5Z1I6|Q5Z1I6_NOCFA	25	43973	13 (1)	10 (1)	Uncharacterized protein OS=Nocardia farcinica (strain IFM 10152) OX=247156 GN=NFA_8600 PE=4 SV=1
420.	tr|Q5Z249|Q5Z249_NOCFA	25	74124	14 (1)	9 (1)	Putative ceramidase OS=Nocardia farcinica (strain IFM

No.	Accession	Score	Mass	Matches	Sequences	Name
						10152) OX=247156 GN=NFA_6470 PE=4 SV=1
421.	tr|Q5Z2V5|Q5Z2V5_NOCFA	25	47262	27 (2)	13 (2)	Uncharacterized protein OS=Nocardia farcinica (strain IFM 10152) OX=247156 GN=NFA_3940 PE=4 SV=1
422.	sp|Q5Z1Q6|RL18_NOCFA	25	14687	11 (1)	6 (1)	50S ribosomal protein L18 OS=Nocardia farcinica (strain IFM 10152) OX=247156 GN=rplR PE=3 SV=1
423.	tr|Q5YRN5|Q5YRN5_NOCFA	25	125251	29 (1)	21 (1)	Ribonucleoside-diphosphate reductase OS=Nocardia farcinica (strain IFM 10152) OX=247156 GN=nrdE PE=3 SV=1
424.	tr|Q5YPK6|Q5YPK6_NOCFA	25	155587 1	170 (1)	138 (1)	Putative non-ribosomal peptide synthetase OS=Nocardia farcinica (strain IFM 10152) OX=247156 GN=NFA_50330 PE=4 SV=1
425.	tr|Q5YZY0|Q5YZY0_NOCFA	25	47771	11 (1)	8 (1)	Sulfate adenylyltransferase subunit 1 OS=Nocardia farcinica (strain IFM 10152) OX=247156 GN=cysN PE=3 SV=1
426.	tr|Q5YZ96|Q5YZ96_NOCFA	25	15025	7 (1)	5 (1)	Uncharacterized protein OS=Nocardia farcinica (strain IFM 10152) OX=247156 GN=NFA_16490 PE=4 SV=1
427.	tr|Q5YY33|Q5YY33_NOCFA	25	13946	10 (1)	3 (1)	Uncharacterized protein OS=Nocardia farcinica (strain IFM 10152) OX=247156 GN=NFA_20620 PE=4 SV=1
428.	sp|Q5YPR6|RL31B_NOCFA	25	9977	8 (1)	2 (1)	50S ribosomal protein L31 type B OS=Nocardia farcinica (strain IFM 10152) OX=247156 GN=rpmE2 PE=3 SV=1
429.	tr|Q5Z3G0|Q5Z3G0_NOCFA	25	185546	22 (1)	17 (1)	Putative polyketide synthase OS=Nocardia farcinica (strain IFM 10152) OX=247156 GN=NFA_1890 PE=4 SV=1
430.	tr|Q5YSA8|Q5YSA8_NOCFA	25	16909	3 (1)	3 (1)	Putative acyltransferase OS=Nocardia farcinica (strain IFM 10152) OX=247156 GN=NFA_40840 PE=4 SV=1
431.	tr|Q5Z3S1|Q5Z3S1_NOCFA	25	39976	23 (1)	10 (1)	Putative reductase/deaminase OS=Nocardia farcinica (strain IFM 10152) OX=247156 GN=NFA_780 PE=4 SV=1
432.	tr|Q5YP41|Q5YP41_NOCFA	25	17161	6 (1)	5 (1)	Uncharacterized protein OS=Nocardia farcinica (strain IFM

No.	Accession	Score	Mass	Matches	Sequences	Name
						10152) OX=247156 GN=NFA_51980 PE=4 SV=1
433.	tr|Q5YT16|Q5YT16_NOCFA	24	31472	12 (1)	5 (1)	Putative acyltransferase OS=Nocardia farcinica (strain IFM 10152) OX=247156 GN=NFA_38270 PE=4 SV=1
434.	tr|Q5YX10|Q5YX10_NOCFA	24	33538	6 (2)	2 (1)	Putative ABC transporter substrate-binding protein OS=Nocardia farcinica (strain IFM 10152) OX=247156 GN=NFA_24340 PE=3 SV=1
435.	tr|Q5YTI8|Q5YTI8_NOCFA	24	22316	9 (1)	5 (1)	Uncharacterized protein OS=Nocardia farcinica (strain IFM 10152) OX=247156 GN=NFA_36550 PE=4 SV=1
436.	tr|Q5Z3D4|Q5Z3D4_NOCFA	24	29704	11 (1)	8 (1)	Putative short chain dehydrogenase OS=Nocardia farcinica (strain IFM 10152) OX=247156 GN=NFA_2150 PE=4 SV=1
437.	tr|Q5YVZ9|Q5YVZ9_NOCFA	24	642800	80 (1)	55 (1)	Putative non-ribosomal peptide synthetase OS=Nocardia farcinica (strain IFM 10152) OX=247156 GN=NFA_27950 PE=4 SV=1
438.	tr|Q5YM88|Q5YM88_NOCFA	24	15851	5 (1)	4 (1)	Uncharacterized protein OS=Nocardia farcinica (strain IFM 10152) OX=247156 GN=PNF2_180 PE=4 SV=1
439.	tr|Q5YU69|Q5YU69_NOCFA	24	37359	23 (2)	10 (2)	Putative oxidoreductase OS=Nocardia farcinica (strain IFM 10152) OX=247156 GN=NFA_34240 PE=4 SV=1
440.	tr|Q5YTR8|Q5YTR8_NOCFA	24	58072	33 (1)	19 (1)	Glucose-6-phosphate 1-dehydrogenase OS=Nocardia farcinica (strain IFM 10152) OX=247156 GN=zwf PE=3 SV=1
441.	tr|Q5YPA9|Q5YPA9_NOCFA	24	151055	34 (2)	18 (2)	Uncharacterized protein OS=Nocardia farcinica (strain IFM 10152) OX=247156 GN=NFA_51300 PE=3 SV=1
442.	tr|Q5YY77|Q5YY77_NOCFA	24	74409	13 (1)	12 (1)	Uncharacterized protein OS=Nocardia farcinica (strain IFM 10152) OX=247156 GN=NFA_20180 PE=4 SV=1
443.	sp|Q5YYF7|ARGB_NOCFA	24	31562	14 (2)	5 (2)	Acetylglutamate kinase OS=Nocardia farcinica (strain IFM 10152) OX=247156 GN=argB PE=3 SV=1

No.	Accession	Score	Mass	Matches	Sequences	Name
444.	sp|Q5Z0Y3|ATPA_NOCFA	23	58690	31 (1)	15 (1)	ATP synthase subunit alpha OS=Nocardia farcinica (strain IFM 10152) OX=247156 GN=atpA PE=3 SV=1
445.	tr|Q5YTG9|Q5YTG9_NOCFA	23	50795	24 (1)	12 (1)	Uncharacterized protein OS=Nocardia farcinica (strain IFM 10152) OX=247156 GN=NFA_36740 PE=4 SV=1
446.	tr|Q5Z0W6|Q5Z0W6_NOCFA	23	94335	30 (1)	19 (1)	Putative glycogen phosphorylase OS=Nocardia farcinica (strain IFM 10152) OX=247156 GN=glgP PE=4 SV=1
447.	tr|Q5YSB5|Q5YSB5_NOCFA	23	43919	13 (2)	8 (2)	Putative uroporphyrinogen III methyltransferase OS=Nocardia farcinica (strain IFM 10152) OX=247156 GN=cysG PE=4 SV=1
448.	sp|Q5YYH7|SYFA_NOCFA	23	37348	24 (1)	13 (1)	Phenylalanine--tRNA ligase alpha subunit OS=Nocardia farcinica (strain IFM 10152) OX=247156 GN=pheS PE=3 SV=1
449.	tr|Q5YMM7|Q5YMM7_NOCFA	23	24467	8 (1)	6 (1)	Uncharacterized protein OS=Nocardia farcinica (strain IFM 10152) OX=247156 GN=PNF1_390 PE=4 SV=1
450.	sp|Q5Z3C0|PATR_NOCFA	23	38738	11 (1)	9 (1)	Putative phenylalanine aminotransferase OS=Nocardia farcinica (strain IFM 10152) OX=247156 GN=pat PE=3 SV=1
451.	tr|Q5YU41|Q5YU41_NOCFA	23	37752	21 (1)	12 (1)	Putative oxidoreductase OS=Nocardia farcinica (strain IFM 10152) OX=247156 GN=NFA_34520 PE=4 SV=1
452.	tr|Q5YPX2|Q5YPX2_NOCFA	23	76800	22 (1)	20 (1)	Uncharacterized protein OS=Nocardia farcinica (strain IFM 10152) OX=247156 GN=NFA_49170 PE=4 SV=1
453.	tr|Q5YS92|Q5YS92_NOCFA	22	8582	5 (1)	4 (1)	Uncharacterized protein OS=Nocardia farcinica (strain IFM 10152) OX=247156 GN=NFA_41000 PE=4 SV=1
454.	tr|Q5YW54|Q5YW54_NOCFA	22	16559	8 (1)	3 (1)	Uncharacterized protein OS=Nocardia farcinica (strain IFM 10152) OX=247156 GN=NFA_27400 PE=4 SV=1
455.	tr|Q5Z2Z7|Q5Z2Z7_NOCFA	22	35080	13 (1)	8 (1)	Putative hydrolase OS=Nocardia farcinica (strain IFM 10152)

No.	Accession	Score	Mass	Matches	Sequences	Name
						OX=247156 GN=NFA_3520 PE=4 SV=1
456.	tr|Q5Z1M1|Q5Z1M1_NOCFA	22	51357	14 (1)	8 (1)	Uncharacterized protein OS=Nocardia farcinica (strain IFM 10152) OX=247156 GN=NFA_8250 PE=4 SV=1
457.	tr|Q5YS40|Q5YS40_NOCFA	22	31254	14 (1)	8 (1)	Putative transcriptional regulator OS=Nocardia farcinica (strain IFM 10152) OX=247156 GN=NFA_41520 PE=4 SV=1
458.	tr|Q5Z0A6|Q5Z0A6_NOCFA	22	74550	23 (1)	14 (1)	Putative 2,4-dienoyl-CoA reductase OS=Nocardia farcinica (strain IFM 10152) OX=247156 GN=NFA_12900 PE=4 SV=1
459.	tr|Q5YM29|Q5YM29_NOCFA	22	32055	10 (1)	6 (1)	Uncharacterized protein OS=Nocardia farcinica (strain IFM 10152) OX=247156 GN=PNF2_740 PE=4 SV=1
460.	tr|Q5Z0B5|Q5Z0B5_NOCFA	22	45538	4 (1)	3 (1)	Putative aminotransferase OS=Nocardia farcinica (strain IFM 10152) OX=247156 GN=NFA_12810 PE=3 SV=1
461.	tr|Q5YNH6|Q5YNH6_NOCFA	22	59411	3 (1)	2 (1)	Uncharacterized protein OS=Nocardia farcinica (strain IFM 10152) OX=247156 GN=NFA_54130 PE=4 SV=1
462.	tr|Q5YYQ1|Q5YYQ1_NOCFA	22	55444	9 (1)	6 (1)	Putative serine/threonine protein kinase OS=Nocardia farcinica (strain IFM 10152) OX=247156 GN=NFA_18440 PE=4 SV=1
463.	tr|Q5YZG4|Q5YZG4_NOCFA	22	31795	8 (1)	4 (1)	Uncharacterized protein OS=Nocardia farcinica (strain IFM 10152) OX=247156 GN=NFA_15810 PE=4 SV=1
464.	tr|Q5YRV0|Q5YRV0_NOCFA	22	35600	16 (1)	7 (1)	Uncharacterized protein OS=Nocardia farcinica (strain IFM 10152) OX=247156 GN=NFA_42420 PE=4 SV=1
465.	tr|Q5YMD5|Q5YMD5_NOCFA	22	32828	7 (2)	4 (1)	Putative DNA-binding protein OS=Nocardia farcinica (strain IFM 10152) OX=247156 GN=PNF1_1310 PE=4 SV=1
466.	tr|Q5YM14|Q5YM14_NOCFA	22	62708	27 (1)	15 (1)	Uncharacterized protein OS=Nocardia farcinica (strain IFM 10152) OX=247156 GN=PNF2_890 PE=4 SV=1
467.	tr|Q5YSD4|Q5YSD4_NOCFA	21	26244	4 (1)	3 (1)	Uncharacterized protein OS=Nocardia farcinica (strain IFM

No.	Accession	Score	Mass	Matches	Sequences	Name
						10152) OX=247156 GN=NFA_40590 PE=4 SV=1
468.	tr|Q5YU34|Q5YU34_NOCFA	21	41385	13 (2)	5 (1)	Putative peptide synthetase OS=Nocardia farcinica (strain IFM 10152) OX=247156 GN=NFA_34590 PE=4 SV=1
469.	tr|Q5Z279|Q5Z279_NOCFA	21	50615	14 (1)	10 (1)	1-acyl-sn-glycerol-3-phosphate acyltransferase OS=Nocardia farcinica (strain IFM 10152) OX=247156 GN=NFA_6170 PE=3 SV=1
470.	tr|Q5Z2N1|Q5Z2N1_NOCFA	21	30391	4 (1)	3 (1)	Uncharacterized protein OS=Nocardia farcinica (strain IFM 10152) OX=247156 GN=NFA_4680 PE=4 SV=1
471.	tr|Q5Z247|Q5Z247_NOCFA	21	15542	9 (1)	7 (1)	Putative transcriptional regulator OS=Nocardia farcinica (strain IFM 10152) OX=247156 GN=NFA_6490 PE=4 SV=1
472.	tr|Q5YMG5|Q5YMG5_NOCFA	21	14663	3 (1)	2 (1)	Uncharacterized protein OS=Nocardia farcinica (strain IFM 10152) OX=247156 GN=PNF1_1010 PE=4 SV=1
473.	sp|Q5Z110|ICMF_NOCFA	20	118338	85 (1)	30 (1)	Fused isobutyryl-CoA mutase OS=Nocardia farcinica (strain IFM 10152) OX=247156 GN=icmF PE=1 SV=1
474.	tr|Q5YSU7|Q5YSU7_NOCFA	20	9450	9 (1)	7 (1)	Uncharacterized protein OS=Nocardia farcinica (strain IFM 10152) OX=247156 GN=NFA_38960 PE=4 SV=1
475.	sp|Q5YTD8|PDXS_NOCFA	20	32758	19 (2)	12 (2)	Pyridoxal 5'-phosphate synthase subunit PdxS OS=Nocardia farcinica (strain IFM 10152) OX=247156 GN=pdxS PE=3 SV=1
476.	tr|Q5YND7|Q5YND7_NOCFA	20	39214	12 (1)	9 (1)	Uncharacterized protein OS=Nocardia farcinica (strain IFM 10152) OX=247156 GN=NFA_54520 PE=4 SV=1
477.	tr|Q5YNN6|Q5YNN6_NOCFA	20	36187	19 (1)	7 (1)	Putative dehydrogenase OS=Nocardia farcinica (strain IFM 10152) OX=247156 GN=NFA_53530 PE=4 SV=1
478.	sp|Q5Z1W3|RL3_NOCFA	20	23254	12 (1)	9 (1)	50S ribosomal protein L3 OS=Nocardia farcinica (strain IFM 10152) OX=247156 GN=rplC PE=3 SV=1

No.	Accession	Score	Mass	Matches	Sequences	Name
479.	tr|Q5YYL7|Q5YYL7_NOCFA	20	13534	3 (1)	3 (1)	Thioredoxin OS=Nocardia farcinica (strain IFM 10152) OX=247156 GN=NFA_18780 PE=3 SV=1
480.	tr|Q5YWK5|Q5YWK5_NOCFA	19	51252	16 (1)	10 (1)	Cytochrome P450 monooxygenase OS=Nocardia farcinica (strain IFM 10152) OX=247156 GN=cyp51 PE=3 SV=1
481.	tr|Q5YYL3|Q5YYL3_NOCFA	19	46129	41 (1)	22 (1)	RNA polymerase sigma factor OS=Nocardia farcinica (strain IFM 10152) OX=247156 GN=NFA_18820 PE=3 SV=1
482.	tr|Q5Z3G1|Q5Z3G1_NOCFA	19	71081	4 (1)	4 (1)	Putative acyl-CoA synthetase OS=Nocardia farcinica (strain IFM 10152) OX=247156 GN=NFA_1880 PE=4 SV=1
483.	tr|Q5YU68|Q5YU68_NOCFA	19	22993	6 (1)	4 (1)	Uncharacterized protein OS=Nocardia farcinica (strain IFM 10152) OX=247156 GN=NFA_34250 PE=4 SV=1
484.	tr|Q5YW36|Q5YW36_NOCFA	19	28936	20 (1)	16 (1)	Putative ABC transporter ATP-binding protein OS=Nocardia farcinica (strain IFM 10152) OX=247156 GN=NFA_27580 PE=4 SV=1
485.	sp|Q5YNA5|TRMB_NOCFA	19	28972	10 (1)	4 (1)	tRNA (guanine-N(7)-)-methyltransferase OS=Nocardia farcinica (strain IFM 10152) OX=247156 GN=trmB PE=3 SV=1
486.	tr|Q5YTY6|Q5YTY6_NOCFA	19	49101	14 (1)	8 (1)	Putative glucarate dehydratase OS=Nocardia farcinica (strain IFM 10152) OX=247156 GN=NFA_35070 PE=3 SV=1
487.	tr|Q5YWB3|Q5YWB3_NOCFA	19	80527	23 (1)	17 (1)	Histidine kinase OS=Nocardia farcinica (strain IFM 10152) OX=247156 GN=NFA_26810 PE=4 SV=1
488.	sp|Q5Z0G1|HUTG_NOCFA	19	32554	7 (1)	5 (1)	Formimidoylglutamase OS=Nocardia farcinica (strain IFM 10152) OX=247156 GN=hutG PE=3 SV=1
489.	tr|Q5YRW0|Q5YRW0_NOCFA	18	69281	15 (1)	10 (1)	Acetolactate synthase OS=Nocardia farcinica (strain IFM 10152) OX=247156 GN=ilvB PE=3 SV=1
490.	tr|Q5Z332|Q5Z332_NOCFA	18	16562	2 (1)	2 (1)	Uncharacterized protein OS=Nocardia farcinica (strain IFM

No.	Accession	Score	Mass	Matches	Sequences	Name
						10152) OX=247156 GN=NFA_3170 PE=4 SV=1
491.	tr|Q5YU28|Q5YU28_NOCFA	18	37618	17 (1)	12 (1)	Putative transporter ATPase/kinase OS=Nocardia farcinica (strain IFM 10152) OX=247156 GN=NFA_34650 PE=4 SV=1
492.	tr|Q5Z094|Q5Z094_NOCFA	18	72079	9 (1)	2 (1)	Uncharacterized protein OS=Nocardia farcinica (strain IFM 10152) OX=247156 GN=NFA_13020 PE=4 SV=1
493.	tr|Q5YMD3|Q5YMD3_NOCFA	18	13620	7 (1)	4 (1)	Uncharacterized protein OS=Nocardia farcinica (strain IFM 10152) OX=247156 GN=PNF1_1330 PE=4 SV=1
494.	tr|Q5YT17|Q5YT17_NOCFA	18	36946	3 (1)	3 (1)	Putative esterase OS=Nocardia farcinica (strain IFM 10152) OX=247156 GN=NFA_38260 PE=4 SV=1
495.	tr|Q5Z139|Q5Z139_NOCFA	18	47785	11 (1)	7 (1)	Putative oxidoreductase OS=Nocardia farcinica (strain IFM 10152) OX=247156 GN=NFA_10070 PE=4 SV=1
496.	tr|Q5YQ27|Q5YQ27_NOCFA	18	33999	1 (1)	1 (1)	Uncharacterized protein OS=Nocardia farcinica (strain IFM 10152) OX=247156 GN=NFA_48620 PE=4 SV=1
497.	tr|Q5YXZ6|Q5YXZ6_NOCFA	18	50161	11 (1)	3 (1)	Putative cation transporter OS=Nocardia farcinica (strain IFM 10152) OX=247156 GN=NFA_20990 PE=4 SV=1
498.	sp|Q5YTJ9|SYA_NOCFA	18	95751	26 (1)	17 (1)	Alanine--tRNA ligase OS=Nocardia farcinica (strain IFM 10152) OX=247156 GN=alaS PE=3 SV=1
499.	tr|Q5Z145|Q5Z145_NOCFA	18	17443	7 (1)	6 (1)	N5-carboxyaminoimidazole ribonucleotide mutase OS=Nocardia farcinica (strain IFM 10152) OX=247156 GN=purE PE=3 SV=1
500.	sp|Q5YUA1|CYSD_NOCFA	18	35465	11 (1)	8 (1)	Sulfate adenylyltransferase subunit 2 OS=Nocardia farcinica (strain IFM 10152) OX=247156 GN=cysD PE=3 SV=1
501.	tr|Q5Z268|Q5Z268_NOCFA	17	25576	4 (1)	2 (1)	Putative translocator OS=Nocardia farcinica (strain IFM 10152) OX=247156 GN=NFA_6280 PE=4 SV=1
502.	tr|Q5YQE3|Q5YQE3_NOCFA	17	24333	12 (1)	8 (1)	Putative transcriptional regulator OS=Nocardia farcinica

No.	Accession	Score	Mass	Matches	Sequences	Name
						(strain IFM 10152) OX=247156 GN=NFA_47460 PE=4 SV=1
503.	tr|Q5YXN0|Q5YXN0_NOCFA	17	56936	3 (1)	3 (1)	Uncharacterized protein OS=Nocardia farcinica (strain IFM 10152) OX=247156 GN=NFA_22140 PE=4 SV=1
504.	tr|Q5YP62|Q5YP62_NOCFA	17	27933	9 (1)	6 (1)	Pyrroline-5-carboxylate reductase OS=Nocardia farcinica (strain IFM 10152) OX=247156 GN=proC PE=3 SV=1
505.	tr|Q5Z090|Q5Z090_NOCFA	17	181232	59 (1)	37 (1)	Putative NAD-dependent glutamate dehydrogenase OS=Nocardia farcinica (strain IFM 10152) OX=247156 GN=NFA_13060 PE=4 SV=1
506.	tr|Q5YX24|Q5YX24_NOCFA	17	51139	10 (1)	7 (1)	Putative flavin-containing monooxygenase OS=Nocardia farcinica (strain IFM 10152) OX=247156 GN=NFA_24200 PE=4 SV=1
507.	tr|Q5Z1N5|Q5Z1N5_NOCFA	17	764467	189 (2)	129 (2)	Uncharacterized protein OS=Nocardia farcinica (strain IFM 10152) OX=247156 GN=NFA_8110 PE=4 SV=1
508.	tr|Q5YSR4|Q5YSR4_NOCFA	17	50712	19 (1)	12 (1)	Putative phage protein OS=Nocardia farcinica (strain IFM 10152) OX=247156 GN=NFA_39290 PE=4 SV=1
509.	tr|Q5YZ72|Q5YZ72_NOCFA	16	83750	28 (1)	14 (1)	Uncharacterized protein OS=Nocardia farcinica (strain IFM 10152) OX=247156 GN=NFA_16730 PE=4 SV=1
510.	tr|Q5Z2F3|Q5Z2F3_NOCFA	16	32376	13 (1)	8 (1)	Uncharacterized protein OS=Nocardia farcinica (strain IFM 10152) OX=247156 GN=NFA_5430 PE=4 SV=1
511.	tr|Q5YT44|Q5YT44_NOCFA	16	14231	14 (1)	1 (1)	Uncharacterized protein OS=Nocardia farcinica (strain IFM 10152) OX=247156 GN=NFA_37990 PE=4 SV=1
512.	tr|Q5YZE4|Q5YZE4_NOCFA	16	75473	11 (1)	10 (1)	Putative para-aminobenzoate synthase OS=Nocardia farcinica (strain IFM 10152) OX=247156 GN=pabB PE=4 SV=1
513.	tr|Q5YZC3|Q5YZC3_NOCFA	16	23505	7 (1)	4 (1)	Putative transcriptional regulator OS=Nocardia farcinica (strain IFM 10152) OX=247156 GN=NFA_16220 PE=4 SV=1

No.	Accession	Score	Mass	Matches	Sequences	Name
514.	tr|Q5Z1X8|Q5Z1X8_NOCFA	16	207672	16 (1)	15 (1)	Putative non-ribosomal peptide synthetase OS=Nocardia farcinica (strain IFM 10152) OX=247156 GN=NFA_7180 PE=4 SV=1
515.	tr|Q5YME0|Q5YME0_NOCFA	16	30643	28 (1)	7 (1)	Uncharacterized protein OS=Nocardia farcinica (strain IFM 10152) OX=247156 GN=PNF1_1260 PE=4 SV=1
516.	tr|Q5YZC9|Q5YZC9_NOCFA	16	10737	3 (1)	3 (1)	Acyl carrier protein OS=Nocardia farcinica (strain IFM 10152) OX=247156 GN=acpM PE=3 SV=1
517.	tr|Q5YNM9|Q5YNM9_NOCFA	16	15035	24 (1)	5 (1)	Uncharacterized protein OS=Nocardia farcinica (strain IFM 10152) OX=247156 GN=NFA_53600 PE=4 SV=1
518.	tr|Q5Z0U5|Q5Z0U5_NOCFA	16	71823	20 (1)	16 (1)	Putative transporter OS=Nocardia farcinica (strain IFM 10152) OX=247156 GN=NFA_11010 PE=3 SV=1
519.	tr|Q5YZ62|Q5YZ62_NOCFA	15	20423	11 (1)	3 (1)	Uncharacterized protein OS=Nocardia farcinica (strain IFM 10152) OX=247156 GN=NFA_16830 PE=4 SV=1
520.	tr|Q5YND8|Q5YND8_NOCFA	15	66246	13 (1)	9 (1)	Putative acyl-CoA dehydrogenase OS=Nocardia farcinica (strain IFM 10152) OX=247156 GN=fadE49 PE=3 SV=1
